# Supplementary material for: Unraveling the electronegativity-dominated intermediate adsorption on high-entropy alloy electrocatalysts
Source: Nat Commun. 2022 May 13;13:2662. doi: 10.1038/s41467-022-30379-4 (PMC9106752; doi:10.1038/s41467-022-30379-4)
Supplement: Supplementary file 1 — Supplementary Information [file 41467_2022_30379_MOESM1_ESM.pdf]

Supplementary information

Unraveling the electronegativity-dominated intermediate adsorption on high-entropy alloy electrocatalysts

Jiace Hao[1,6], Zechao Zhuang[2,6], Kecheng Cao[3], Guohua Gao[4], Chan Wang[1], Feili Lai[5], Shuanglong Lu[1], Piming Ma[1], Weifu Dong[1], Tianxi Liu[1], Mingliang Du[1], Han Zhu[1]*

[1] Key Laboratory of Synthetic and Biological Colloids, Ministry of Education, School of Chemical and Material Engineering, Jiangnan University, Wuxi 214122, China.

[2] Department of Chemistry, Tsinghua University, Beijing 100084, P. R. China.

[3] School of Physical Science and Technology, ShanghaiTech University, Shanghai 201210, P. R. China.

[4] Department of Chemistry, KU Leuven, Celestijnenlaan 200F, Leuven 3001, Belgium.

[5] Shanghai Key Laboratory of Special Artificial Microstructure Materials and Technology, Key Laboratory of Road and Traffic Engineering of the Ministry of Education, Tongji University, Shanghai 200092, P. R. China.

[6] These authors contributed equally: Jiace Hao and Zechao Zhuang. Correspondence and requests for materials should be addressed to H. Z. (email: zhysw@jiangnan.edu.cn)

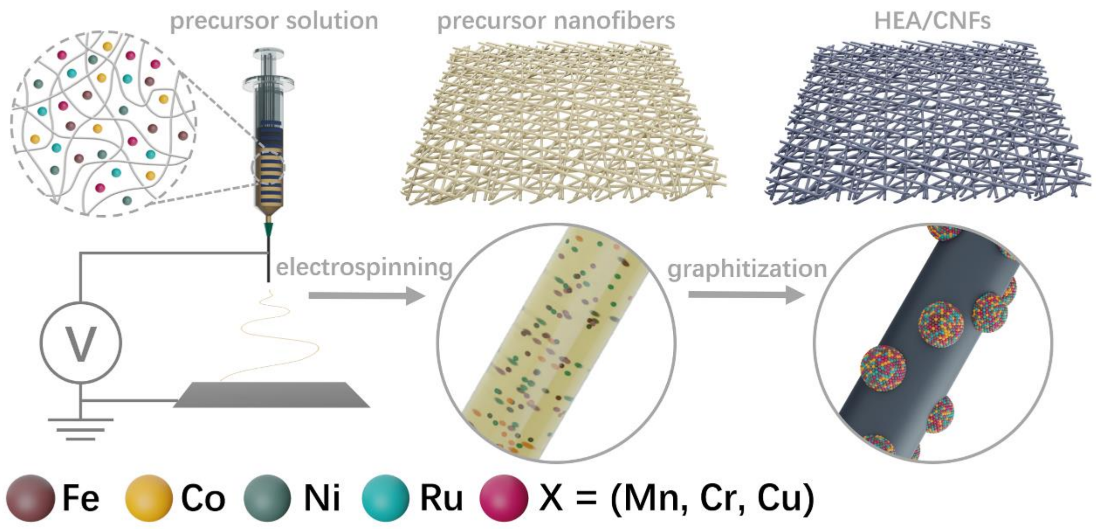

Supplementary Fig. 1. Schematic illustration for the synthetic processes of the FeCoNiXRu/CNFs (X=Mn, Cr and Cu).

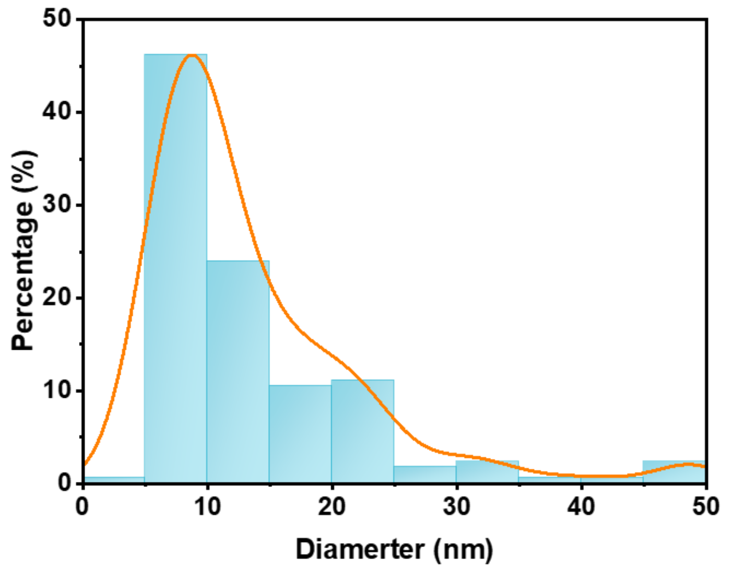

Supplementary Fig. 2. Average diameters of the FeCoNiMnRu HEA NPs supported on CNFs.

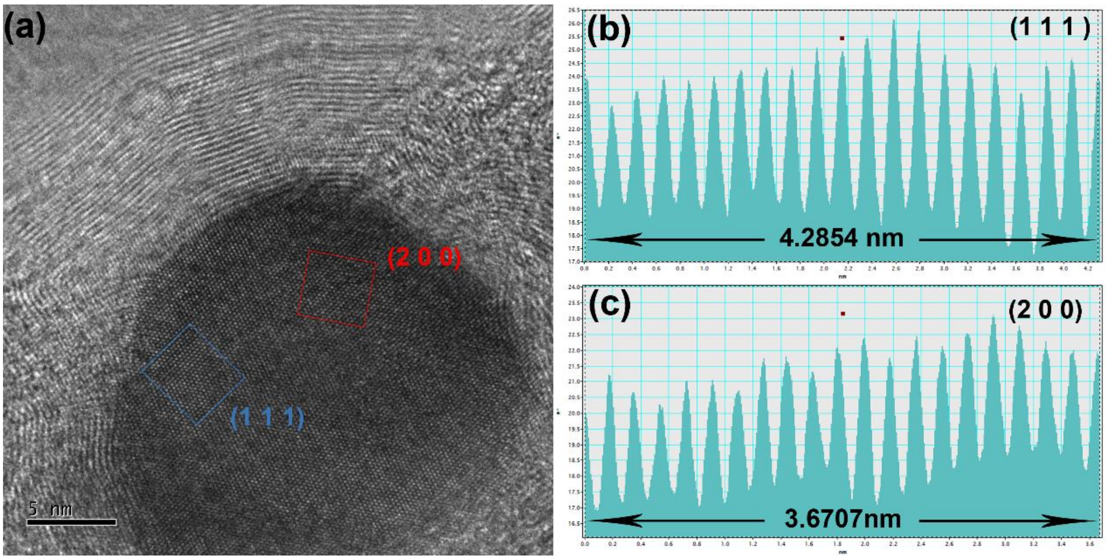

Supplementary Fig. 3. (a) HRTEM image of the FeCoNiMnRu HEA NP supported on CNFs. (b, c) Intensity profile recorded from the area indicated by the quadrate box in panel.

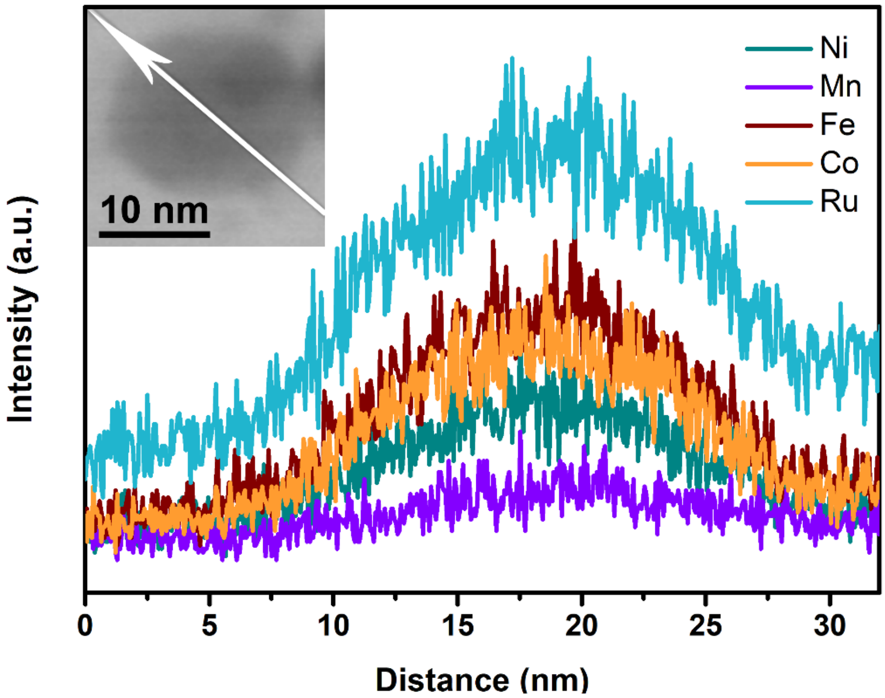

Supplementary Fig. 4. Line-scan STEM-EDS spectra of a FeCoNiMnRu NP supported on CNFs.

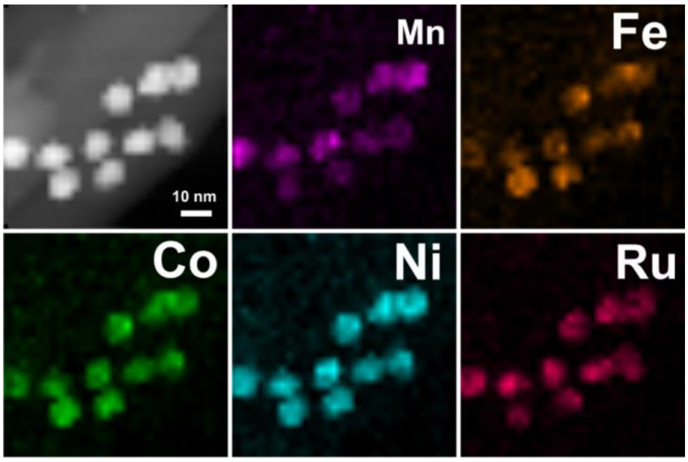

Supplementary Fig. 5. STEM-EDS mapping of the HEA NPs supported on CNFs.

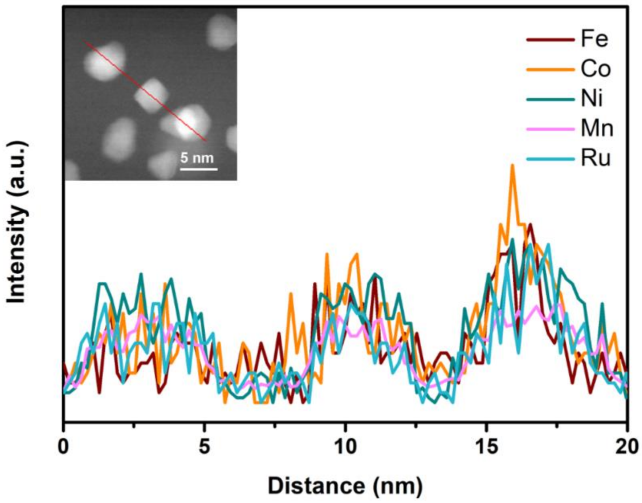

Supplementary Fig. 6. Line-scan STEM-EDX spectra of the HEA NPs supported on CNFs.

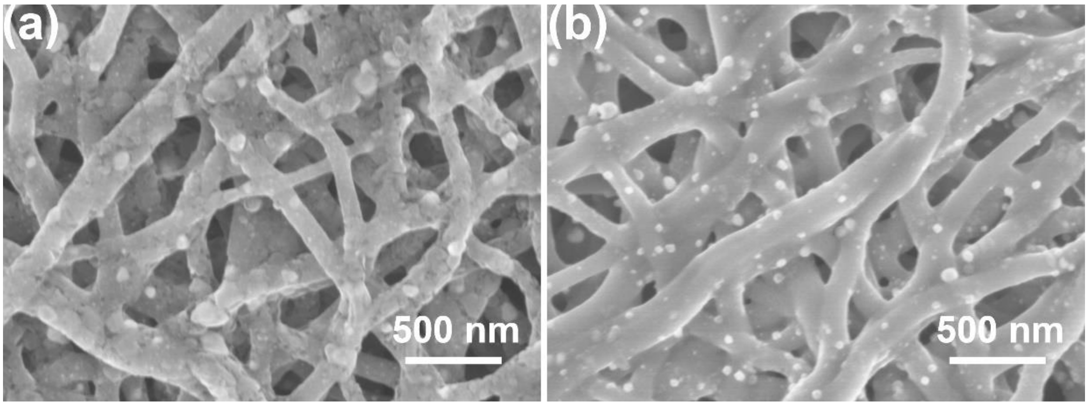

Supplementary Fig. 7. FE-SEM images of the (a) FeCoNiCrRu/CNFs and (b) FeCoNiCuRu/CNFs.

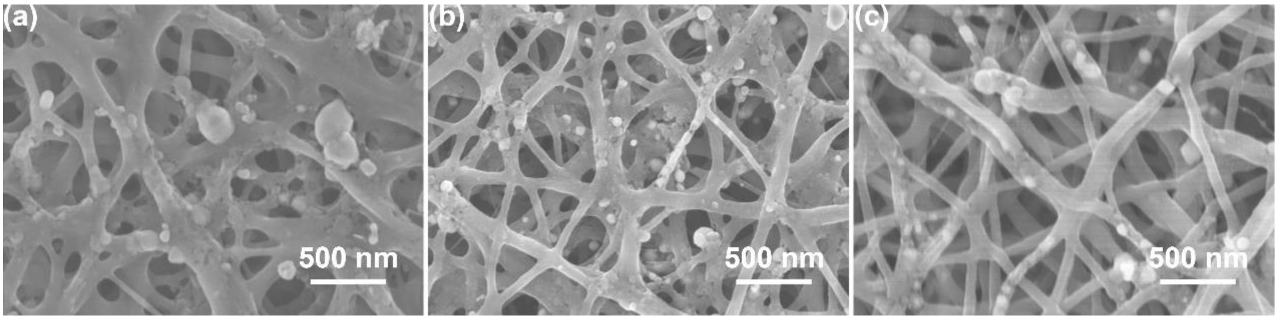

Supplementary Fig. 8. FE-SEM images of the as-prepared (a) FeCoNi/CNFs, (b) FeCoNiMn/CNFs, and (c) FeCoNiRu/CNFs.

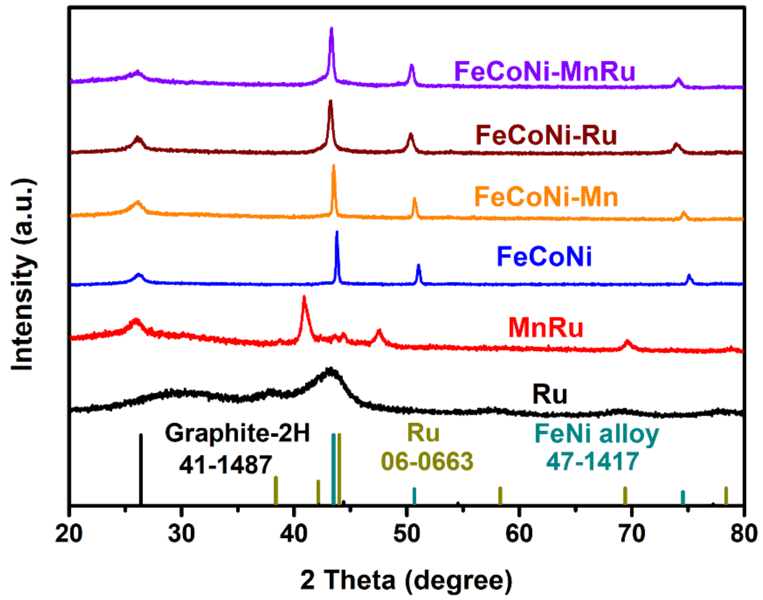

Supplementary Fig. 9. XRD patterns of FeCoNi/CNFs, FeCoNiMn/CNFs, and FeCoNiRu/CNFs.

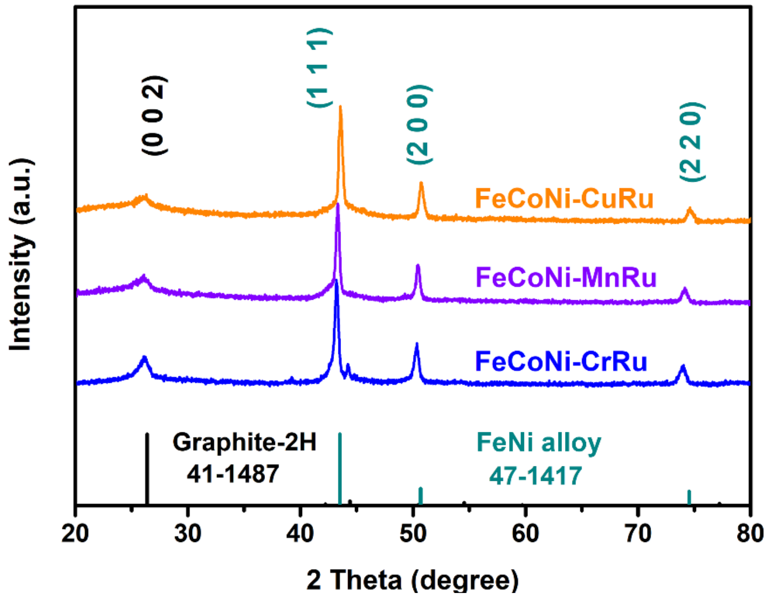

Supplementary Fig. 10. XRD patterns of FeCoNiMnRu/CNFs, FeCoNiCrRu/CNFs, and FeCoNiCuRu/CNFs.

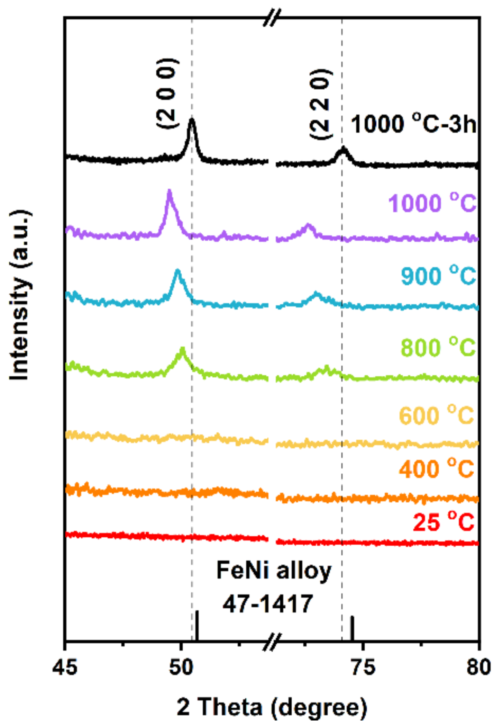

Supplementary Fig. 11. The enlarged *in situ* XRD patterns of FeCoNiMnRu/CNFs showing the peak shift of (200) and (220) planes.

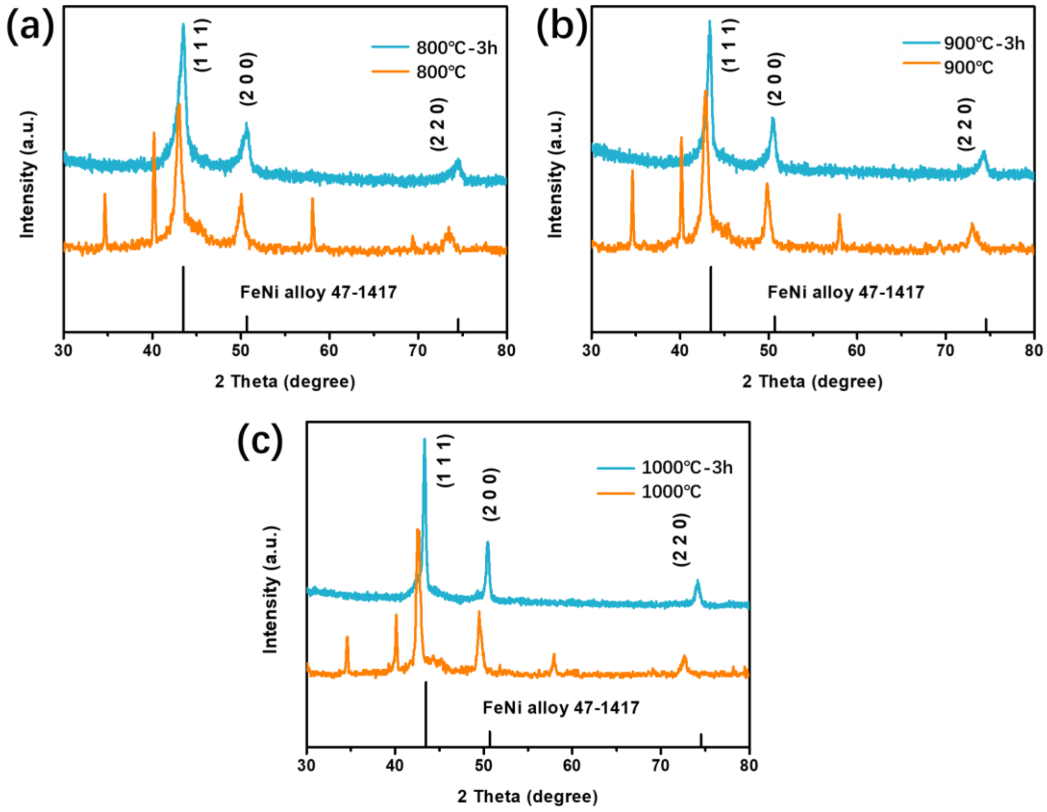

Supplementary Fig. 12. XRD patterns of FeCoNiMnRu/CNFs obtained after prolonged heat treatment (upper) and during *in situ* characterization (lower) at (a) 800 °C, (b) 900 °C, and 1000 °C.

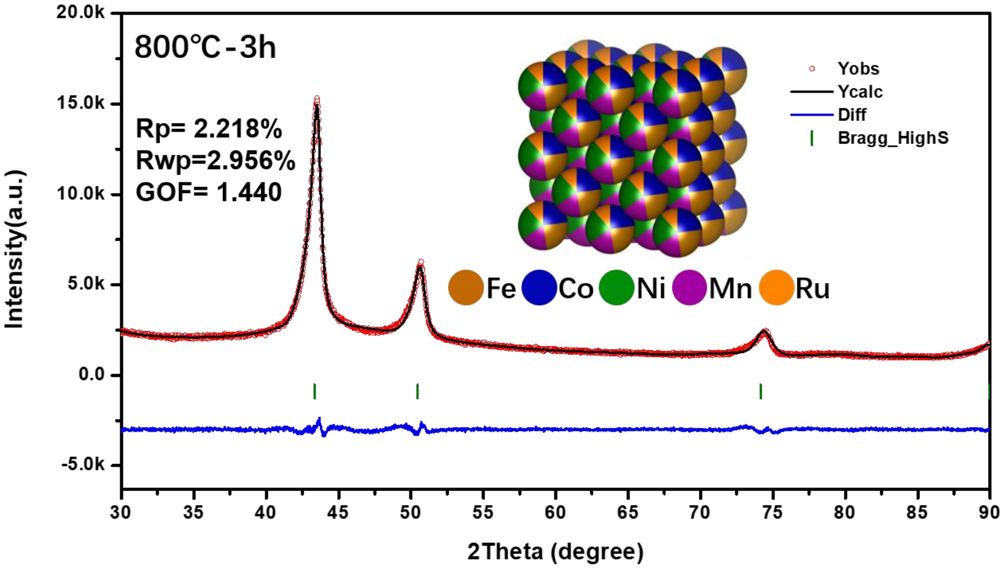

Supplementary Fig. 13. XRD pattern of FeCoNiMnRu/CNFs (800 ºC-3 h) with a scan rate of 0.5°/min and the corresponding XRD Rietveld refinement.

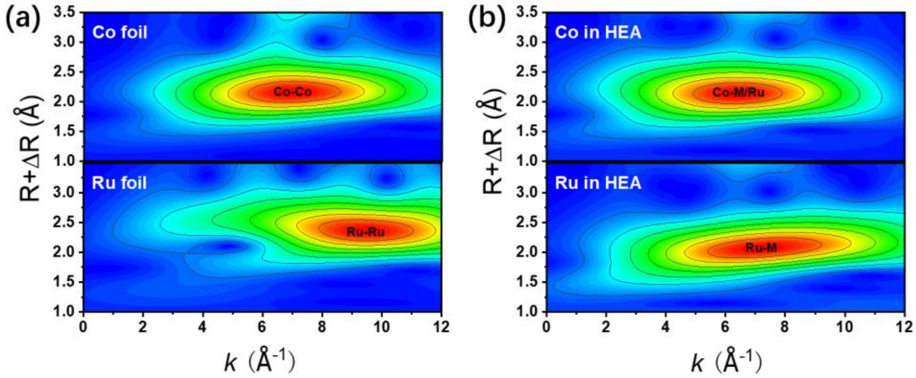

Supplementary Figure 14. WT-EXAFS spectra of (a) Co and Ru foil, (b) Co and Ru in FeCoNiMnRu/CNFs.

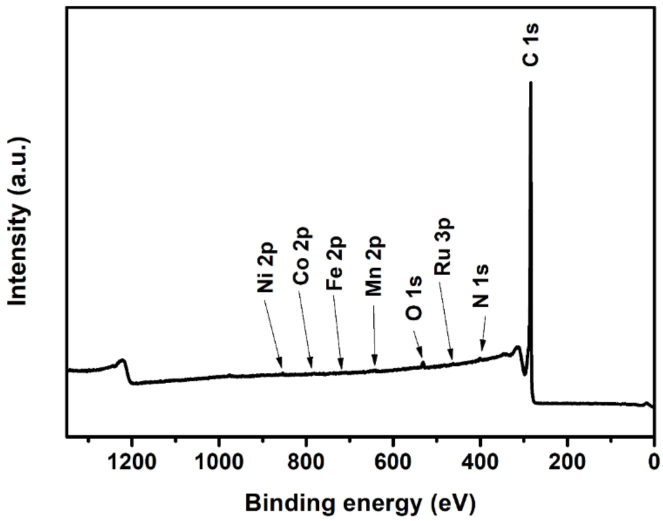

Supplementary Fig. 15. XPS survey spectrum of the as-prepared FeCoNiMnRu/CNFs.

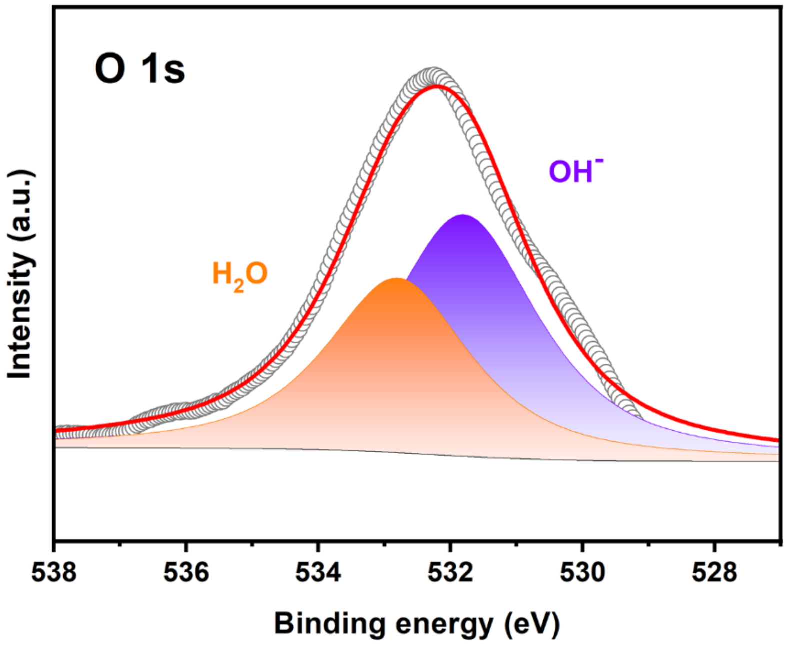

Supplementary Fig. 16. High-resolution O 1s XPS spectrum of FeCoNiMnRu/CNFs.

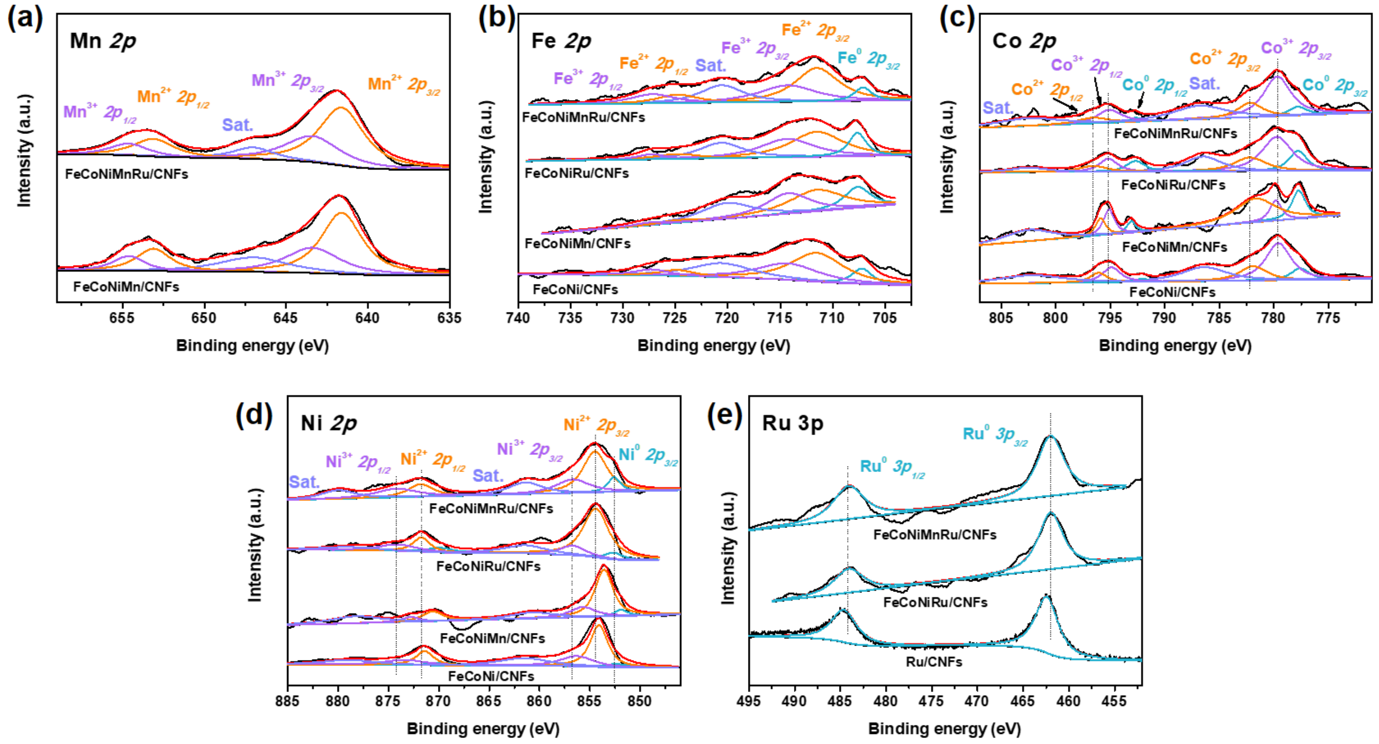

Supplementary Fig. 17. High-resolution (a) Mn 2p XPS spectrum of FeCoNiMn/CNFs and FeCoNiMnRu/CNFs, (b) Fe 2P, (c) Co 2p, (d) Ni 2p XPS spectra of FeCoNi/CNFs, FeCoNiMn/CNFs, FeCoNiRu/CNFs, and FeCoNiMnRu/CNFs, (e) Ru 3p XPS spectrum of Ru/CNFs, FeCoNiRu/CNFs, and FeCoNiMnRu/CNFs.

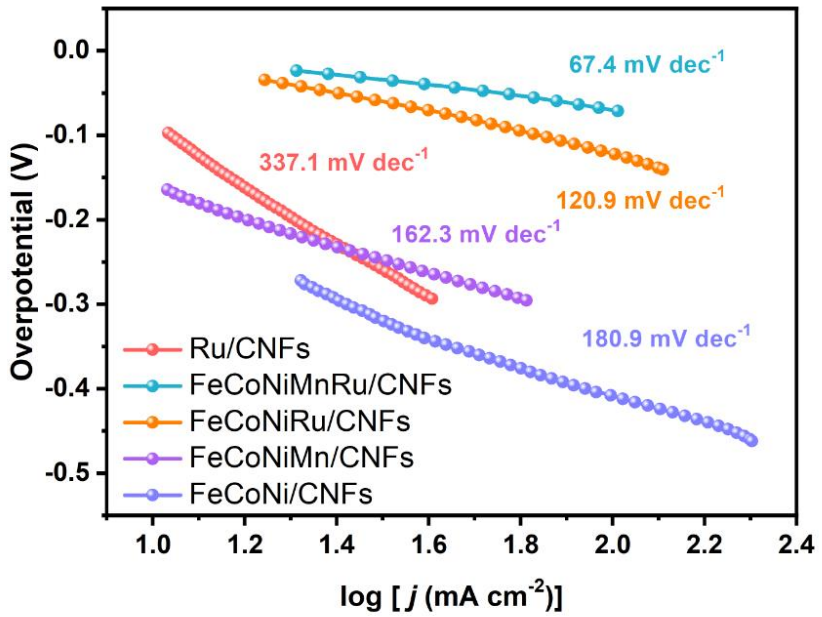

Supplementary Fig. 18. Tafel slopes of the as-prepared electrocatalysts for HER.

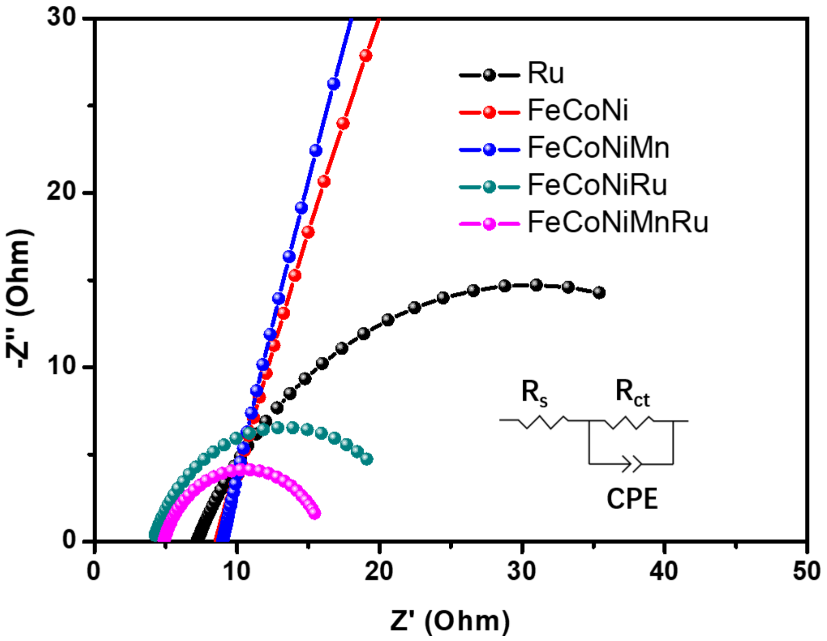

Supplementary Fig. 19. EIS spectra of the as-prepared electrocatalysts.

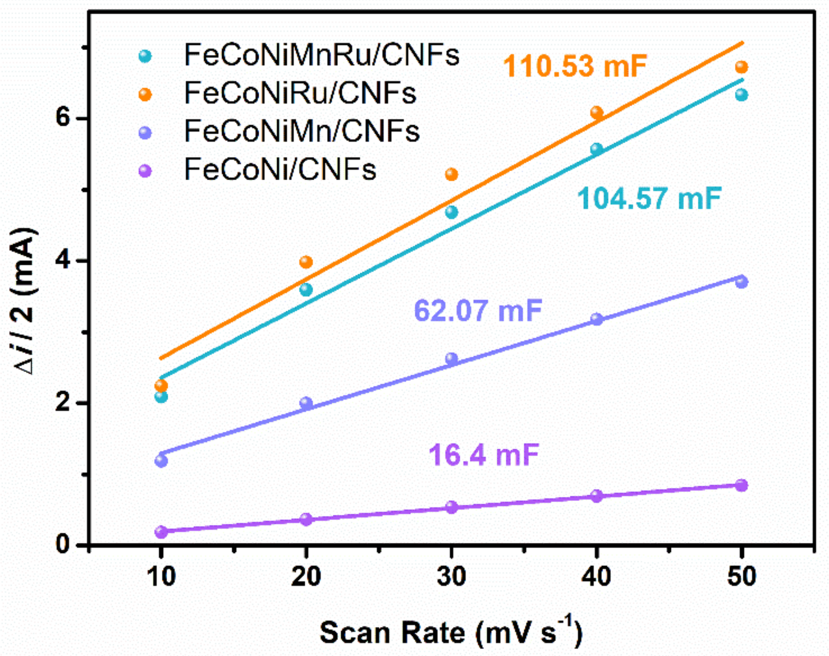

Supplementary Fig. 20. Double-layer capacitance per geometric area (C_{dl}) of the as-prepared electrodes.

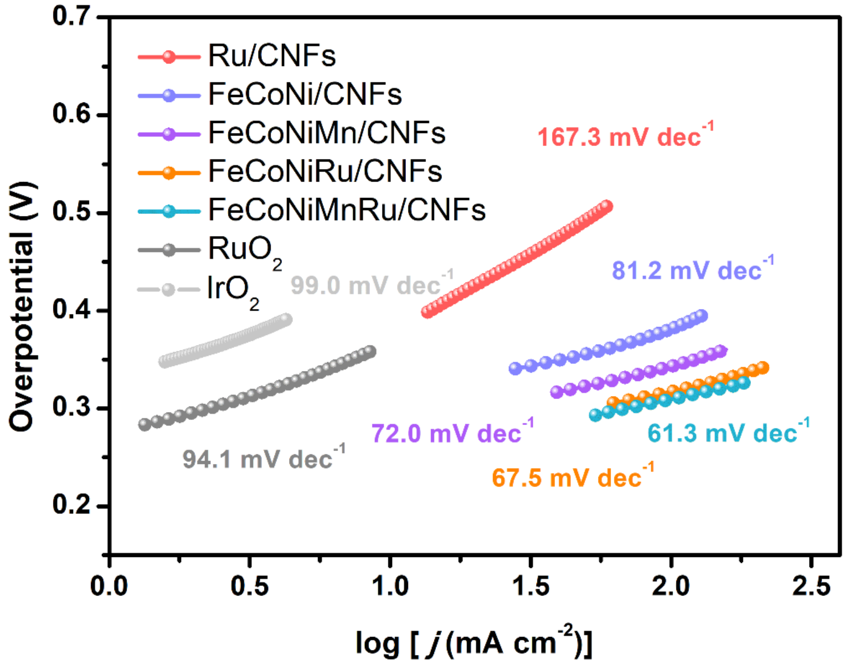

Supplementary Fig. 21. Tafel slopes of the as-prepared electrocatalysts for OER.

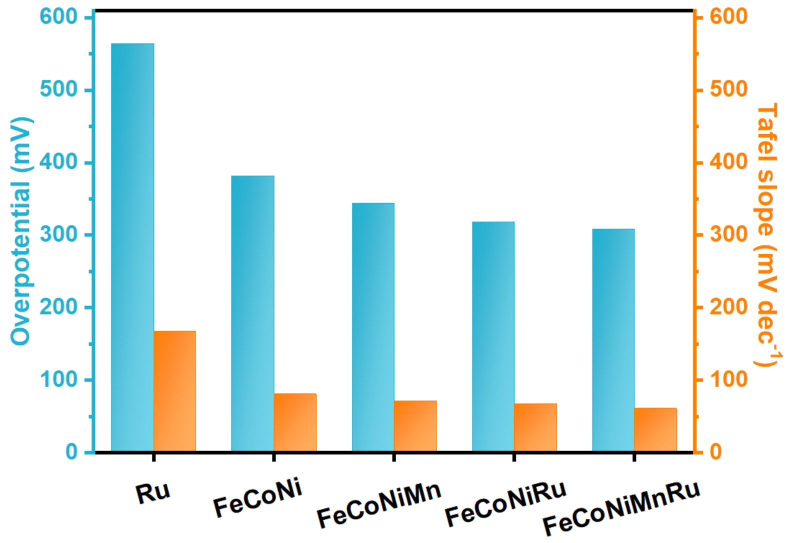

Supplementary Fig. 22. The corresponding histogram of OER overpotentials at 100 mA cm^{-2} and Tafel slopes obtained on FeCoNi/CNFs, FeCoNiMn/CNFs, FeCoNiRu/CNFs, FeCoNiMnRu/CNFs, and Ru/C in 1.0 M KOH electrolyte.

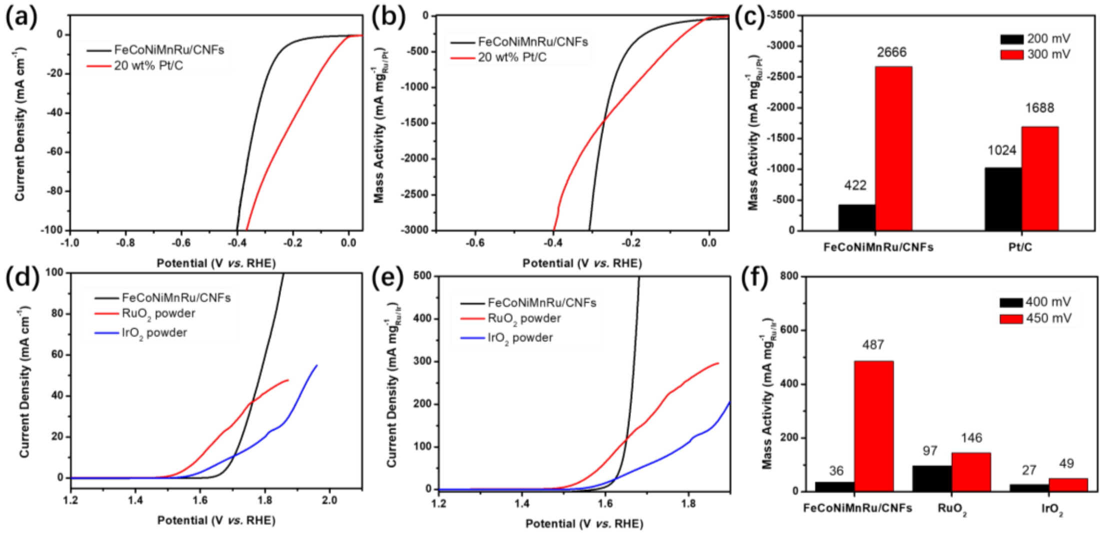

Supplementary Fig. 23. LSV curves of GCE supported FeCoNiMnRu/CNFs and 20 wt% Pt/C for HER normalized by (a) geometric area and (b) mass loading of noble metal (Ru or Pt). (c) The mass activities of FeCoNiMnRu/CNFs and 20 wt% Pt/C for HER at overpotentials of 200 and 300 mV. LSV curves of GCE supported FeCoNiMnRu/CNFs, RuO_2 powder, and IrO_2 powder for OER normalized by (d) geometric area and (e) mass loading of noble metal (Ru or Ir). (f) The mass activities of FeCoNiMnRu/CNFs, RuO_2 powder, and IrO_2 powder for OER at overpotentials of 400 and 450 mV.

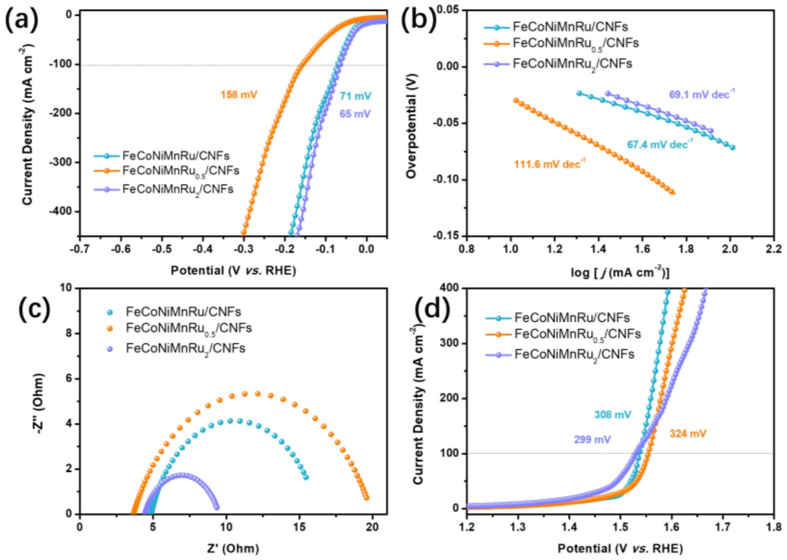

Supplementary Fig. 24. (a) LSV curves, corresponding (b) Tafel plots, and (c) EIS spectra of the as-prepared FeCoNiMnRu/CNFs, FeCoNiMnRu$_{0.5}$/CNFs and FeCoNiMnRu$_2$/CNFs used for HER in 1 M KOH. (d) LSV curves of the as-prepared FeCoNiMnRu/CNFs, FeCoNiMnRu$_{0.5}$/CNFs and FeCoNiMnRu$_2$/CNFs used for OER in 1 M KOH.

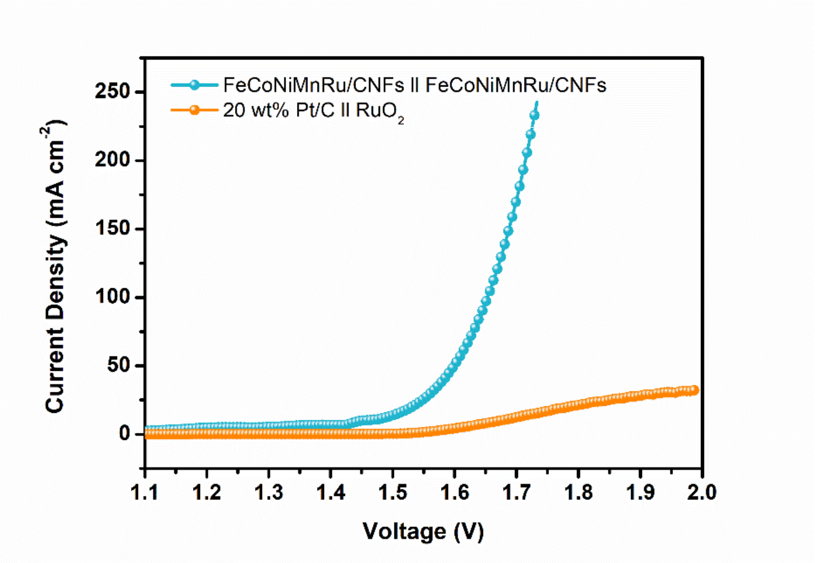

Supplementary Fig. 25. Polarization curves for overall water splitting by FeCoNiMnRu/CNFs ll FeCoNiMnRu/CNFs and Pt/C ll RuO_2 in a two-electrode configuration in 1 M KOH at a scan rate of 2 mV/s.

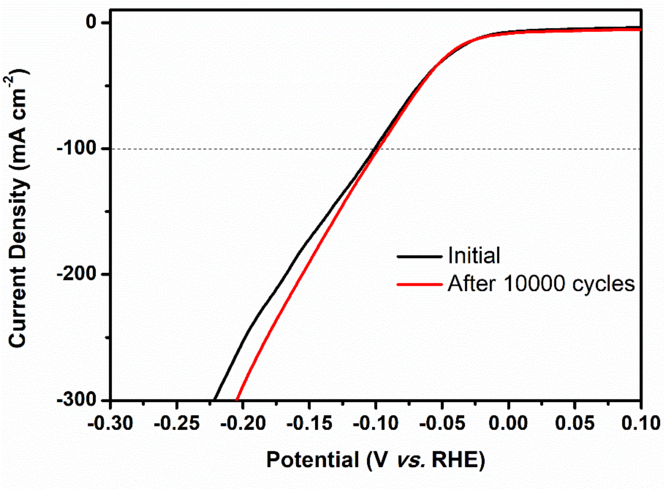

Supplementary Fig. 26. LSV curves of FeCoNiMnRu/CNFs before and after 10000 CV cycles.

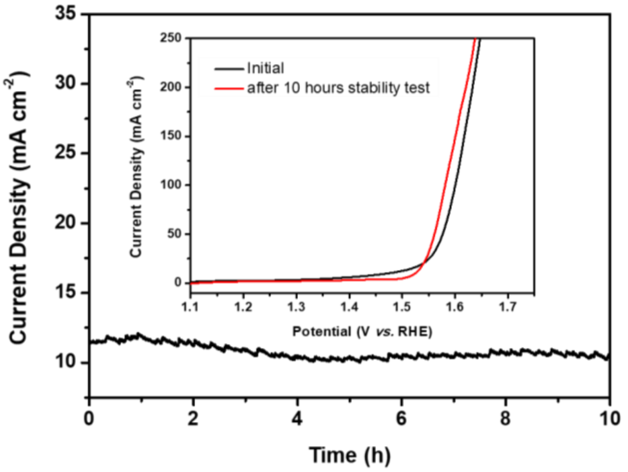

Supplementary Fig. 27. The chronoamperometric curve for FeCoNiMnRu/CNFs measured at 1.55 V vs. RHE for 10 h. Inset shows the LSV curves of FeCoNiMnRu/CNFs before and after stability test.

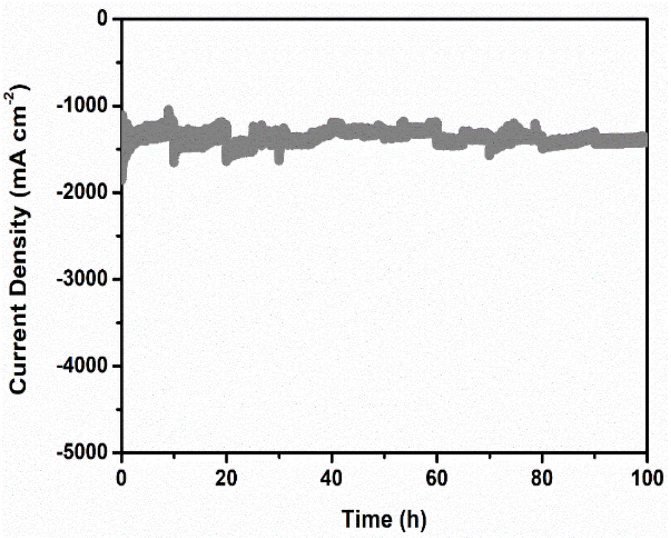

Supplementary Fig. 28. The chronoamperometric curve for FeCoNiMnRu/CNFs measured at 60 °C and -2.22 V vs. RHE for 100 h in 1 M KOH.

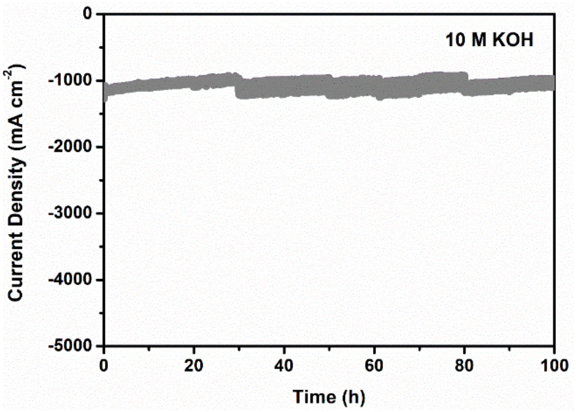

Supplementary Fig. 29. The chronoamperometric curve for FeCoNiMnRu/CNFs measured at -0.77 V vs. RHE for 100 h in 10 M KOH.

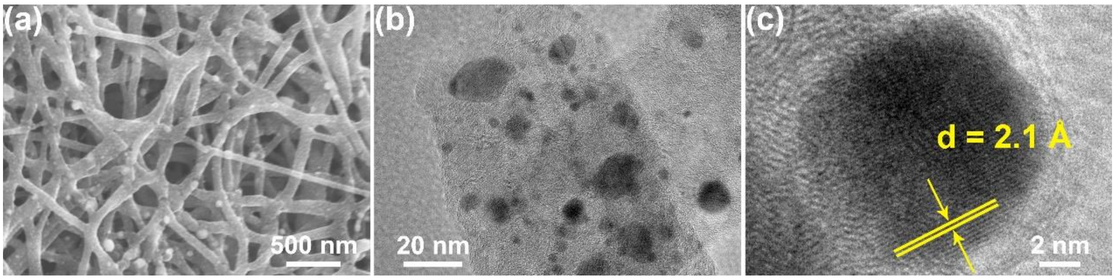

Supplementary Fig. 30. (a) FE-SEM, (b) TEM and (c) HRTEM images of FeCoNiMnRu/CNFs electrode after long-term stability test.

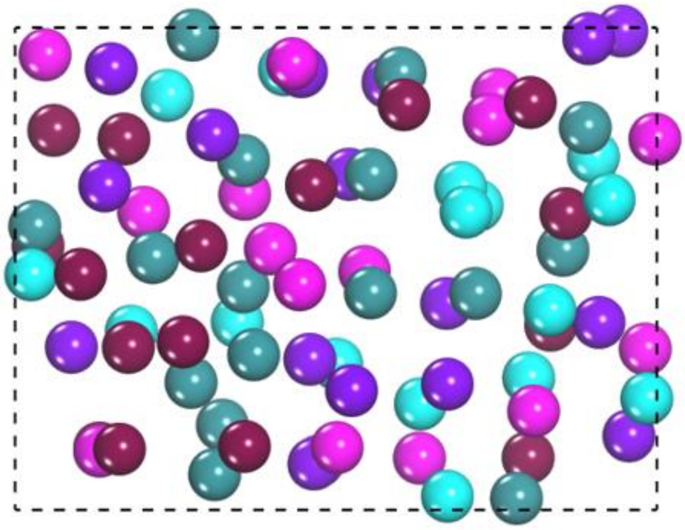

Supplementary Fig. 31. Chemical structure of FeCoNiMnRu HEA.

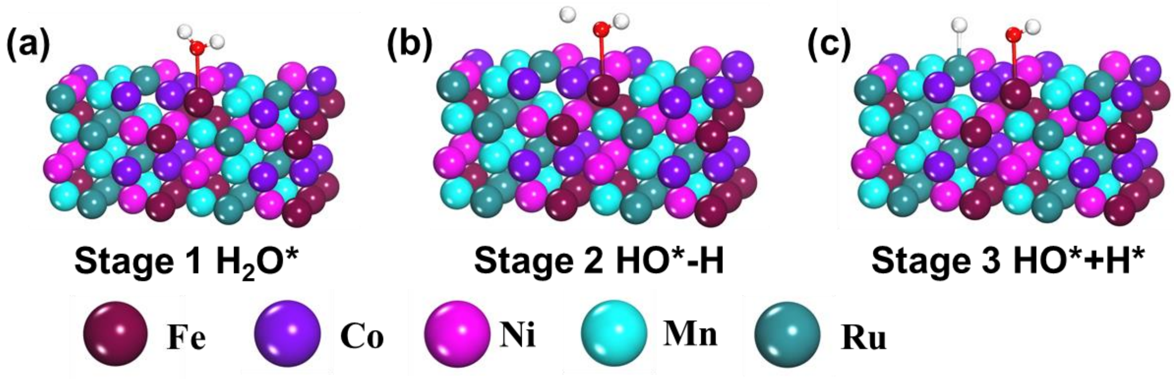

Supplementary Fig. 32. Atomic configurations on Fe site of FeCoNiMnRu HEA at the three stages (a-c) during the H_2O dissociation into OH* and H*.

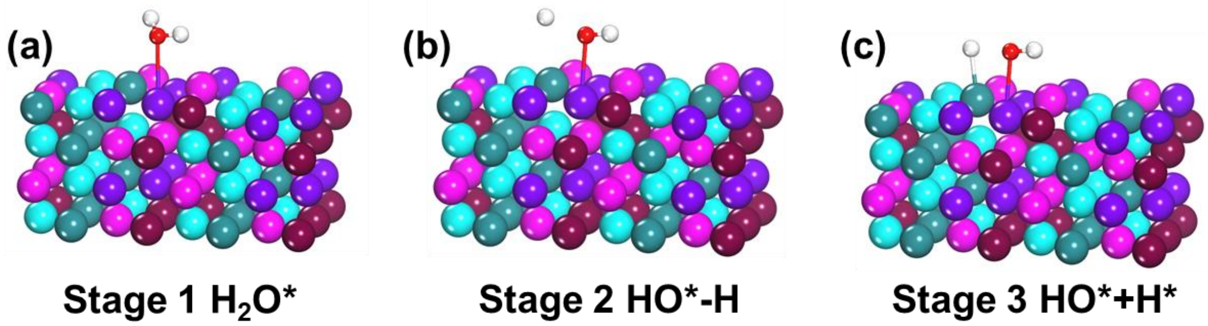

Supplementary Fig. 33. Atomic configurations on Co site of FeCoNiMnRu HEA at the three stages (a-c) during the H_2O dissociation into OH* and H*.

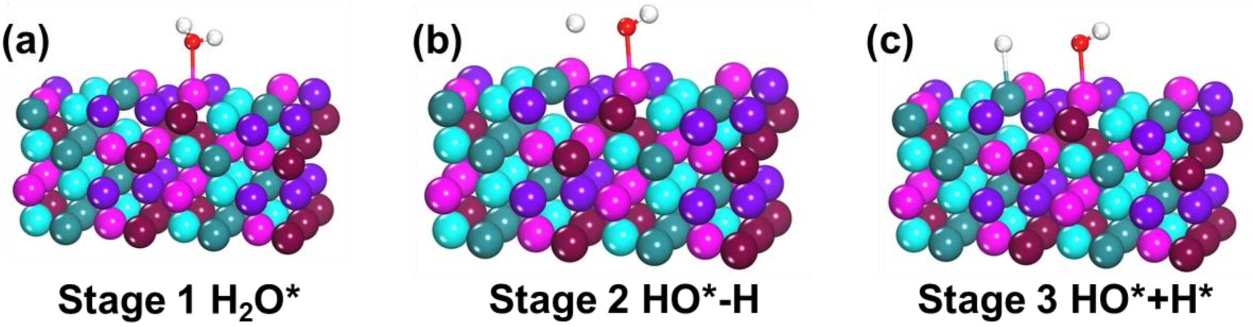

Supplementary Fig. 34. Atomic configurations on Ni site of FeCoNiMnRu HEA at the three stages (a-c) during the H_2O dissociation into OH* and H*.

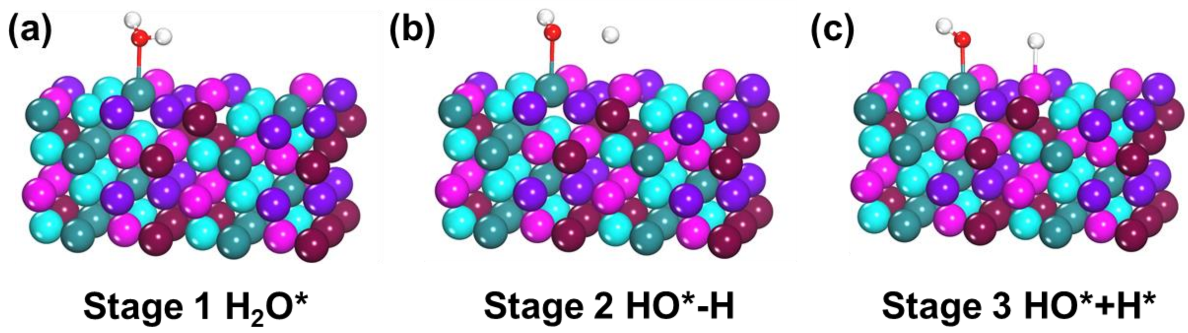

Supplementary Fig. 35. Atomic configurations on Ru site of FeCoNiMnRu HEA at the three stages (a-c) during the H_2O dissociation into OH* and H*.

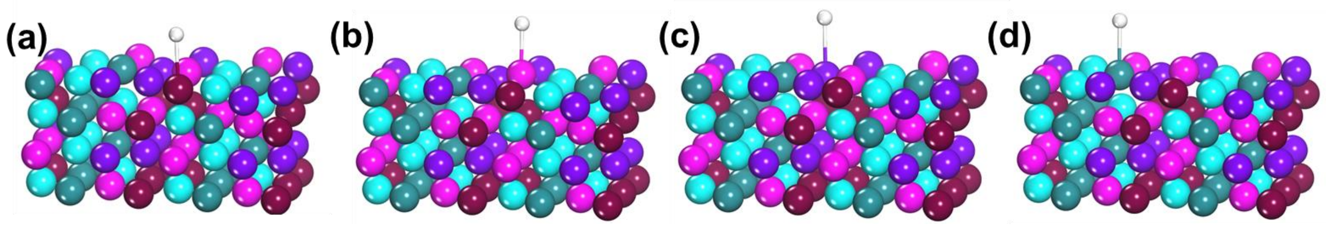

Supplementary Fig. 36. Atomic configurations on (a) Fe, (b) Ni, (c) Co, and (d) Ru sites of FeCoNiMnRu HEA at the stage of H* absorption.

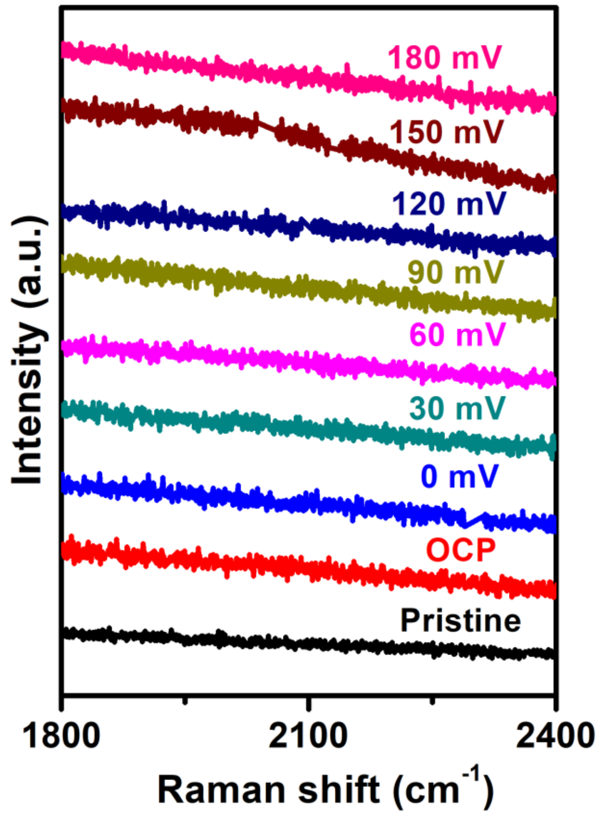

Supplementary Fig. 37. Operando electrochemical-Raman spectra collected by FeCoNiMn/CNFs during the HER process in 1.0 M electrolyte.

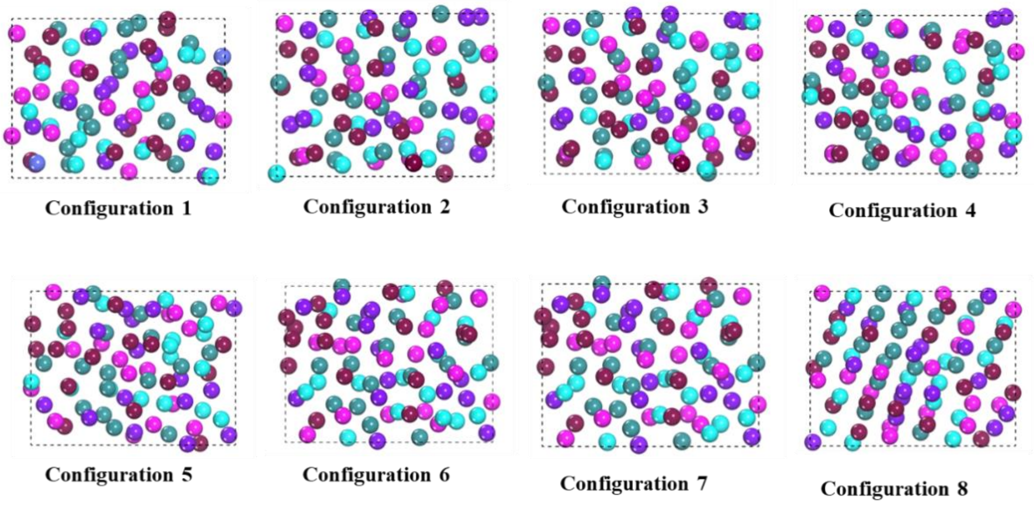

Supplementary Fig. 38. Different atomic configurations of FeCoNiMnRu HEA.

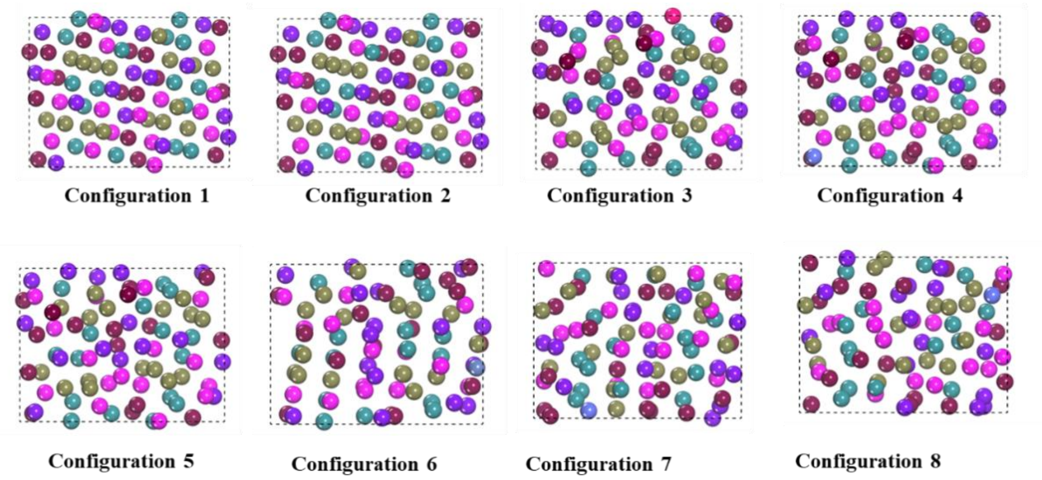

Supplementary Fig. 39. Different atomic configurations of FeCoNiCrRu HEA.

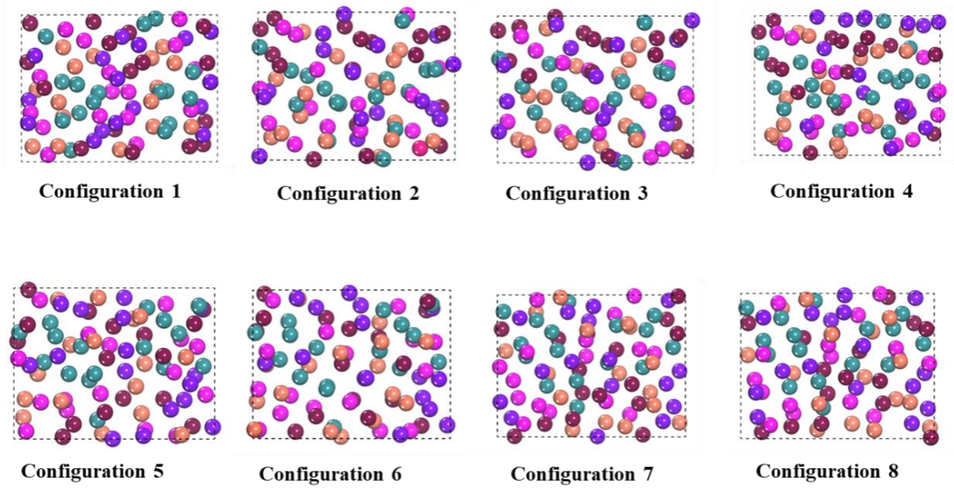

Supplementary Fig. 40. Different atomic configurations of FeCoNiCuRu HEA.

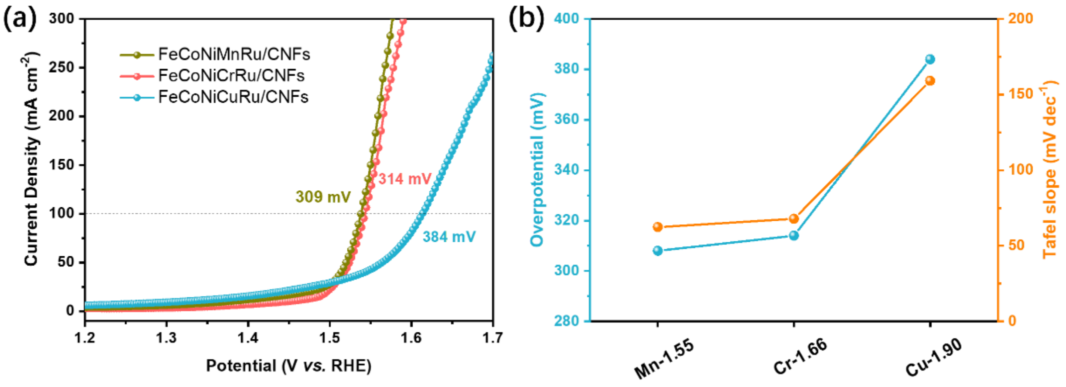

Supplementary Fig. 41. (a) OER polarization curves obtained for FeCoNiXRu/CNFs (X=Cr, Mn, and Cu) in 1.0 M KOH electrolyte. (b) Correlation between the OER overpotentials at 100 mA cm-2, Tafel slopes and the electronegativities of metals X (Cr, Mn, and Cu).

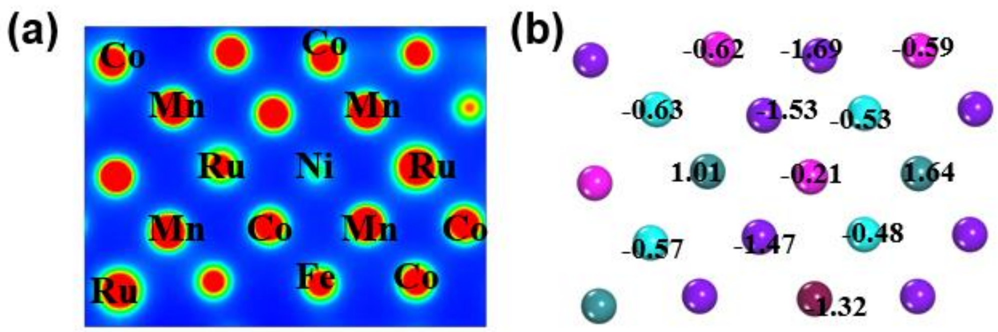

Supplementary Fig. 42. (a) Charge density difference analysis and (b) Bader charge analysis of FeCoNiMnRu system.

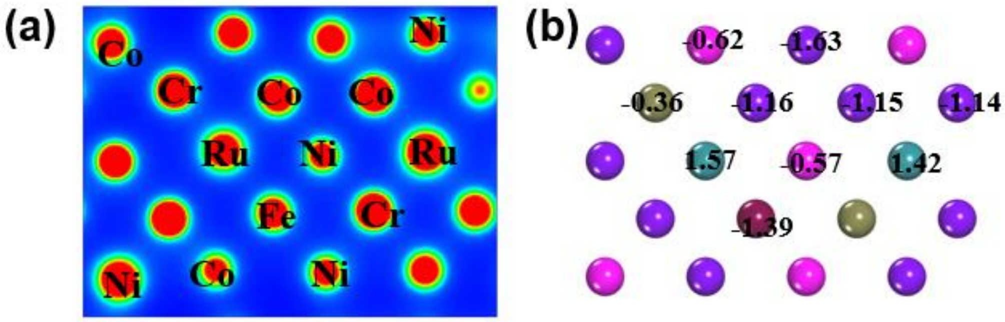

Supplementary Fig. 43. (a) Charge density difference analysis and (b) Bader charge analysis of FeCoNiCrRu system.

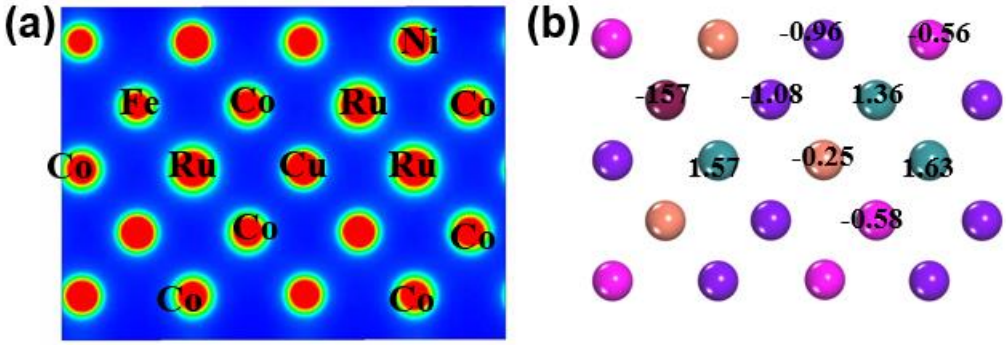

Supplementary Fig. 44. (a) Charge density difference analysis and (b) Bader charge analysis of FeCoNiCuRu system.

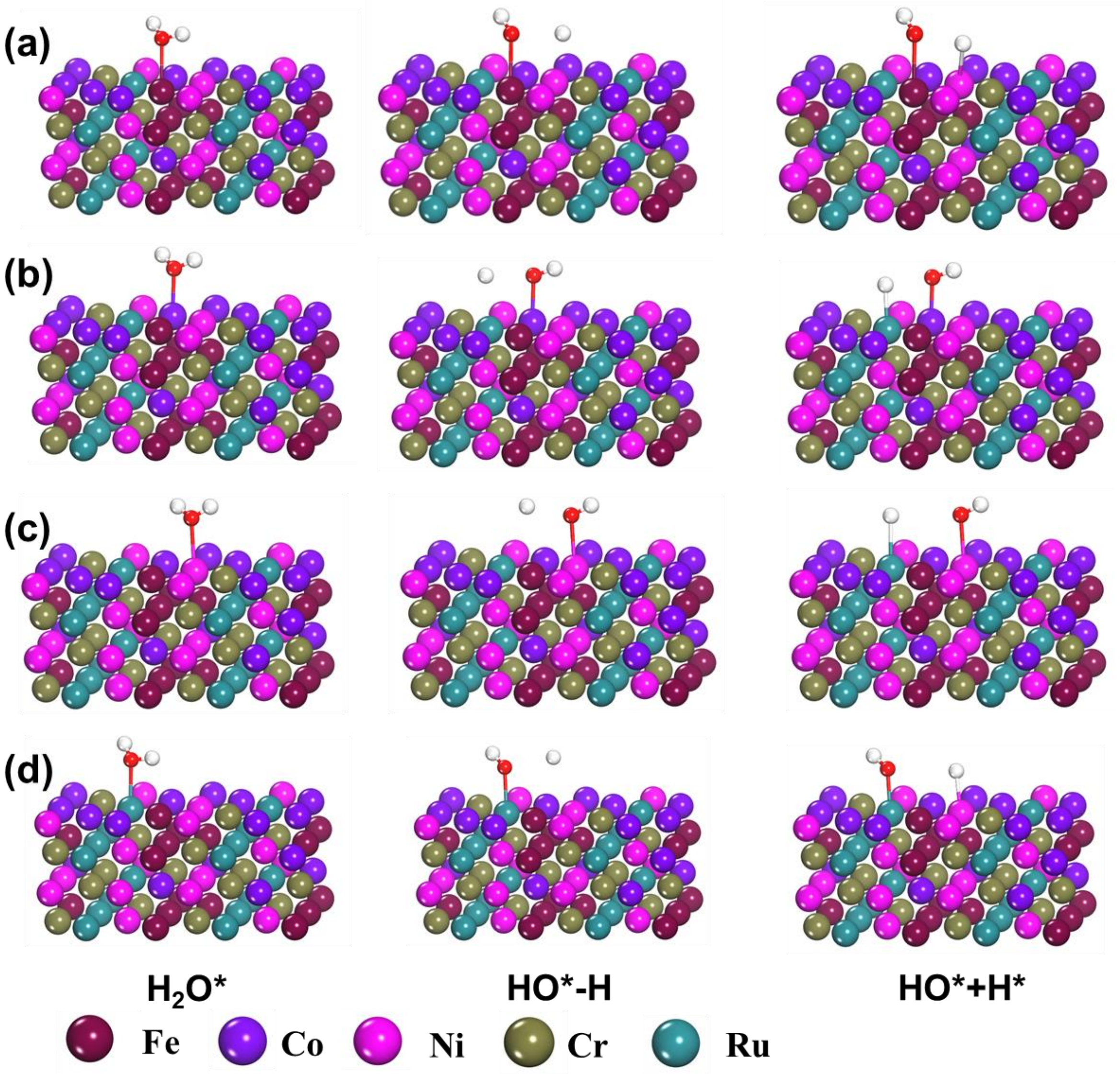

Supplementary Fig. 45. The atomic configurations on (a) Fe, (b) Co, (c) Ni, and (d) Ru catalytic sites of FeCoNiCrRu HEA during the water dissociation.

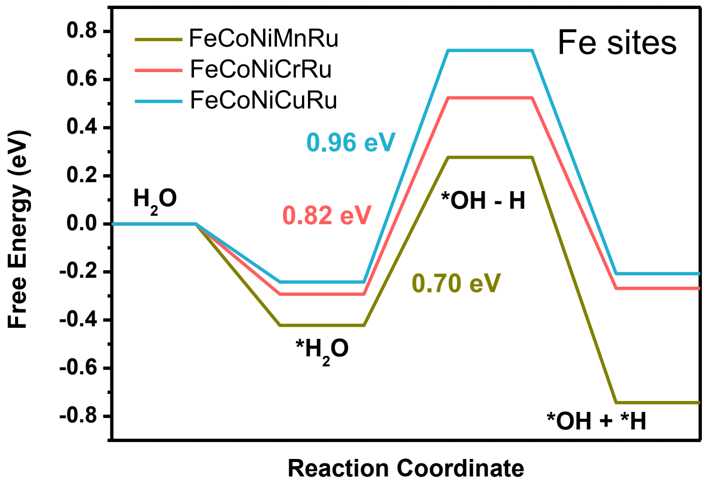

Supplementary Fig. 46. Reaction energy profile of water dissociation on Fe sites of FeCoNiMnRu, FeCoNiCrRu, and FeCoNiCuRu HEA surfaces.

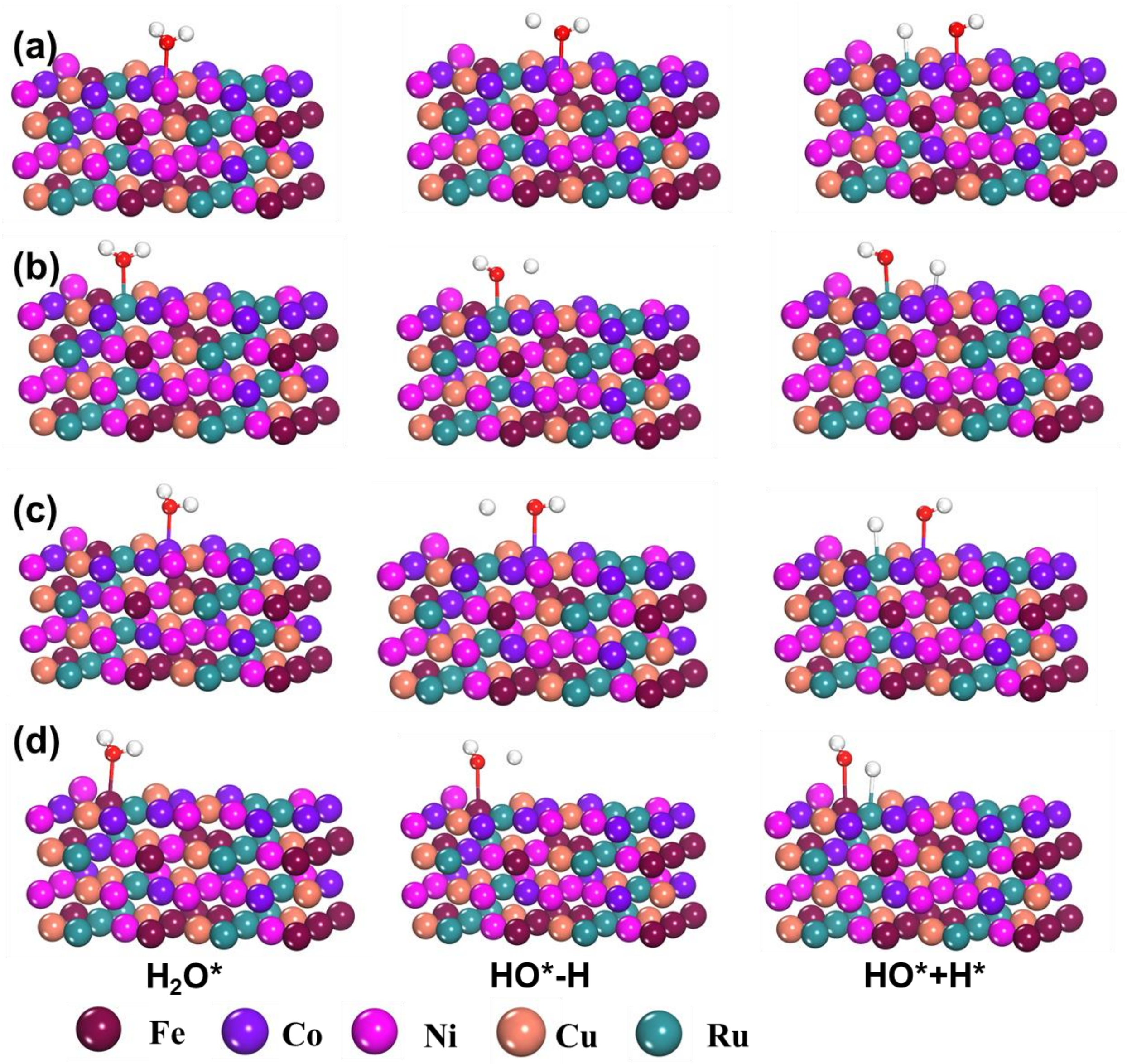

Supplementary Fig. 47. The atomic configurations on (a) Fe, (b) Co, (c) Ni, and (d) Ru catalytic sites of FeCoNiCuRu HEA during the water dissociation.

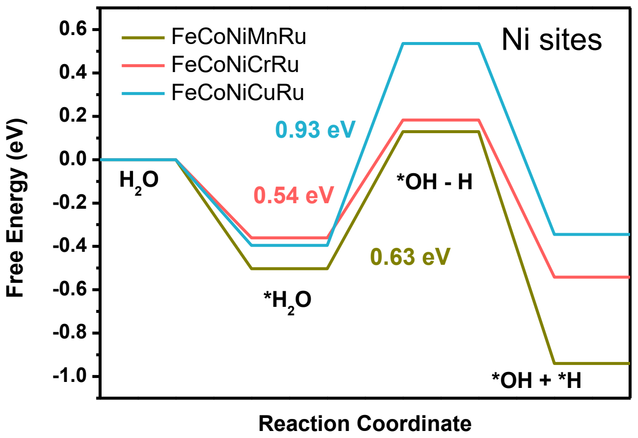

Supplementary Fig. 48. Reaction energy profile of water dissociation on Ni sites of FeCoNiMnRu, FeCoNiCrRu, and FeCoNiCuRu HEA surfaces.

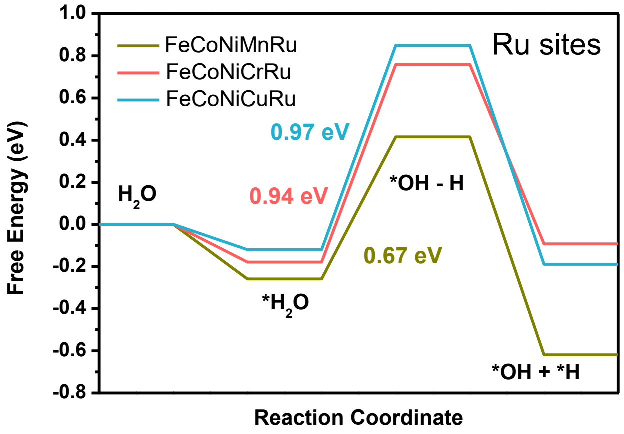

Supplementary Fig. 49. Reaction energy profile of water dissociation on Ru sites of FeCoNiMnRu, FeCoNiCrRu, and FeCoNiCuRu HEA surfaces.

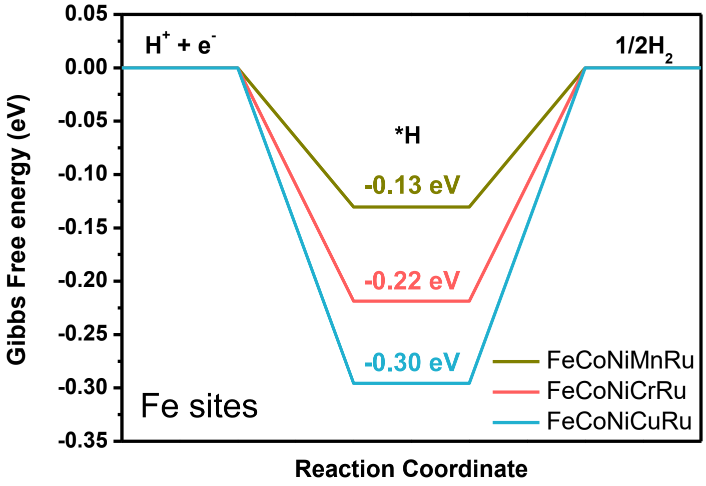

Supplementary Fig. 50. Gibbs free energy (ΔG_{H*}) profiles on Fe sites of FeCoNiMnRu, FeCoNiCrRu, and FeCoNiCuRu HEA surfaces.

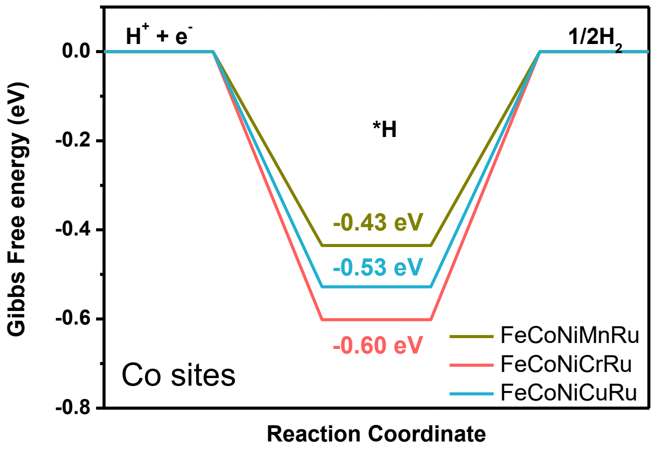

Supplementary Fig. 51. Gibbs free energy (ΔG_{H*}) profiles on Co sites of FeCoNiMnRu, FeCoNiCrRu, and FeCoNiCuRu HEA surfaces.

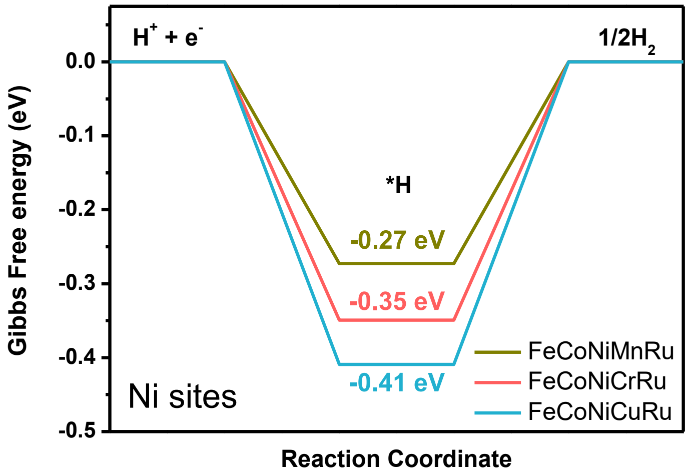

Supplementary Fig. 52. Gibbs free energy (ΔG_{H*}) profiles on Ni sites of FeCoNiMnRu, FeCoNiCrRu, and FeCoNiCuRu HEA surfaces.

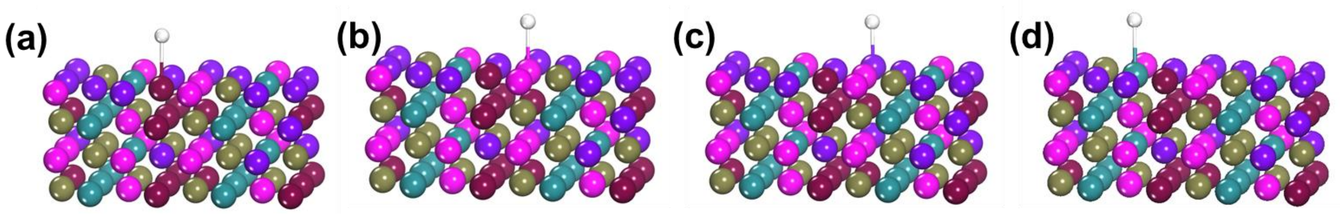

Supplementary Fig. 53. The atomic configurations on (a) Fe, (b) Ni, (c) Co, and (d) Ru catalytic sites of FeCoNiCrRu for H* absorption.

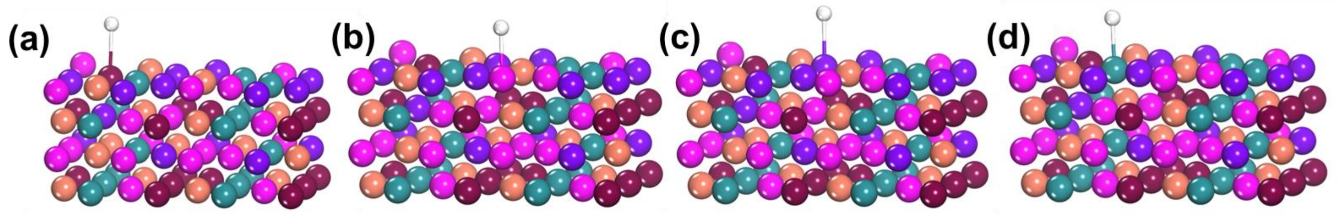

Supplementary Fig. 54. The atomic configurations on (a) Fe, (b) Ni, (c) Co, and (d) Ru catalytic sites of FeCoNiCuRu for H* absorption.

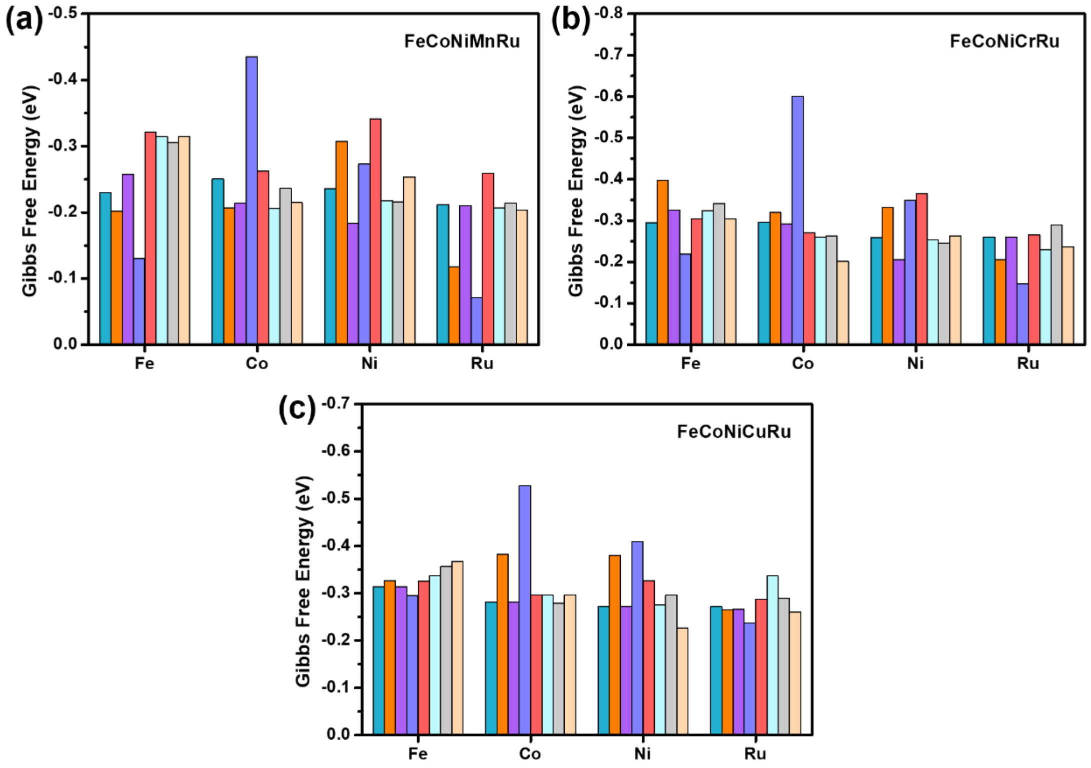

Supplementary Fig. 55. The summarized values of ΔG_{H*} on Fe-, Co-, Ni-, and Ru-sites in (a) FeCoNiMnRu, (b) FeCoNiCrRu, and (c) FeCoNiCuRu with different atomic configurations.

Supplementary Table 1. The mass loadings of Fe, Co, Ni, Mn, and Ru in FeCoNiMnRu/CNFs measured by ICP-OES and the corresponding atomic percentages.

FeCoNiMnRu	Fe	Co	Ni	Mn	Ru
Mass loading (wt. %)	2.97	3.05	2.96	1.78	4.38
Atomic percentage (at. %)	23.00	22.38	21.82	14.03	18.77

Supplementary Table 2. The mass loadings of Fe, Co, Ni, Cr, and Ru in FeCoNiCrRu/CNFs measured by ICP-OES and the corresponding atomic percentages.

FeCoNiMnRu	Fe	Co	Ni	Cr	Ru
Mass loading (wt. %)	2.57	2.82	2.67	2.68	4.07
Atomic percentage (at. %)	19.92	20.71	19.67	22.29	17.41

Supplementary Table 3. The mass loadings of Fe, Co, Ni, Cu, and Ru in FeCoNiCuRu/CNFs measured by ICP-OES and the corresponding atomic percentages.

FeCoNiMnRu	Fe	Co	Ni	Cu	Ru
Mass loading (wt. %)	2.85	2.95	2.57	2.54	4.13
Atomic percentage (at. %)	22.63	22.21	19.38	17.68	18.10

Supplementary Table 4. EXAFS fitting parameters at the Co and Ru K-edge for various samples.

Sample	Shell	*CN*[a]	*R*(Å)[b]	σ^2(Å^2)[c]	ΔE_0(eV)[d]	*R* factor
			Co K-edge (S_0^2=0.811)			
Co foil	Co-Co	12*	2.49	0.0063	6.9	0.0023
CoO	Co-O	6.0	2.10	0.0106	1.5	0.0053
	Co-Co	12.0	3.01	0.0080		
Co_3O_4	Co-O	6.2	1.91	0.0035	-8.1	0.0079
	Co-Co	6.1	2.86	0.0048		
	Co-Co	7.4	3.36	0.0048		
FeCoNiMnRu/CNFs before stability test	Co-Co	7.2	2.51	0.0066	4.5	0.0059
	Co-Ru	1.4	2.49	0.0066		
FeCoNiMnRu/CNFs after stability test	Co-Co	6.9	2.50	0.0085	3.0	0.0071
	Co-Ru	0.3	2.52	0.0085		
			Ru K-edge (S_0^2=0.816)			
Ru foil	Ru-Ru	12*	2.67	0.0033	6.6	0.0071
RuO_2	Ru-O	6.0	1.98	0.0035	2.4	0.0085
	Ru-Ru	3.0	3.12	0.0045		
	Ru-Ru	11.7	3.56	0.0045		

FeCoNiMn Ru/CNFs before stability test	Ru-Co	6.7	2.52	0.0049	-3.0	0.0059

Supplementary Table 5. The summarized binding energies for Ru/CNFs, FeCoNi/CNFs, FeCoNiMn/CNFs, FeCoNiRu/CNFs, and FeCoNiMnRu/CNFs.

	Ru/CNFs	FeCoNi/CNFs	FeCoNiMn/CNFs	FeCoNiRu/CNFs	FeCoNiMnRu/CNFs
Mn^{2+} $2p_{3/2}$	/	/	641.6	/	641.6
Mn^{2+} $2p_{1/2}$	/	/	653.1	/	653.1
Mn^{3+} $2p_{3/2}$	/	/	643.4	/	643.4
Mn^{3+} $2p_{1/2}$	/	/	654.6	/	654.6
Fe^{0} $2p_{3/2}$	/	707.2	707.6	707.6	707.1
Fe^{2+} $2p_{3/2}$	/	711.5	711.5	711.5	711.5
Fe^{2+} $2p_{1/2}$	/	724.8	724.8	724.8	724.8
Fe^{3+} $2p_{3/2}$	/	714.3	714.3	714.3	714.3
Fe^{3+} $2p_{1/2}$	/	727.3	727.3	727.3	727.2
Co^{0} $2p_{3/2}$	/	777.6	777.7	777.8	777.8
Co^{0} $2p_{1/2}$	/	792.1	793.1	792.7	792.7
Co^{2+} $2p_{3/2}$	/	781.9	781.7	782.2	782.2
Co^{2+} $2p_{1/2}$	/	796.1	759.9	796.6	796.6
Co^{3+} $2p_{3/2}$	/	779.6	779.8	779.7	779.7
Co^{3+} $2p_{1/2}$	/	794.9	795.1	795.2	795.1
Ni^{0} $2p_{3/2}$	/	852.4	851.9	852.6	852.6
Ni^{0} $2p_{1/2}$	/	869.7	/	869.9	869.9
Ni^{2+} $2p_{3/2}$	/	854.1	853.6	854.4	854.5
Ni^{2+} $2p_{1/2}$	/	871.4	870.6	871.7	871.8
Ni^{3+} $2p_{3/2}$	/	856.3	855.7	856.7	856.7
Ni^{3+} $2p_{1/2}$	/	873.4	872.8	874.1	874.2
Ru^{0} $3p_{3/2}$	462.4	/	/	462.0	462.0
Ru^{0} $3p_{1/2}$	484.6	/	/	484.1	484.1

Supplementary Table 6. Summarized overpotentials at 10 mA cm^{-2} (η_{10}) and Tafel slopes of electrocatalysts for HER in 1.0 M KOH.

Electrocatalysts	η_{10} (mV)	Tafel slope (mV dec^{-1})	Ref
**FeCoNiMnRu/CNFs**	**5**	**67.4**	**This work**
CuAlNiMoFe	9.7	60	1
FeCoNiAlTi	88.2	40.1	2
NiFeMoCoCr	172	66	3
Ru/CoO NRs	55	72	4
FeNiCoCrMn-G	210	105	5
PdPtCuNiP	32	37.4	6
(Ru–Co)O_x	44.1	23.5	7
Ru_1Ni_1-NCNFs	35	30	8
RFNOH-10	13	30.0	9
RuO_2/NiO/NF	22	31.7	10
Ru-NiFe-P	44	80	11
Ru-MoS_2/CC	41	114	12
Ru-NiCo-LDH	28	42	13
NiFe-MOF-5	163	139	14
Ru/NF-2	10	34	15
ECM@Ru	83	59	16
ES-Ru-ZIF-900	21	64	17
Ru/PC	21	46.6	18
Ru–RuO_2/MoO_3 CNRs-350	9.2	37	19
Ru/$Ni(OH)_2$/NF	25	47	20
$Ru_{1.0}$/NF	47	60	21
Ru@NGT/Ni foam	45	81	22
Ru-Co_3O_4-NiO-NF	44	53.9	23
Ru-CoV-LDH/NF	32	36.4	24

Supplementary Table 7. Summarized overpotentials at 10 mA cm^{-2} (η_{10}) and Tafel slopes of electrocatalysts for OER in 1.0 M KOH.

Electrocatalysts	η_{10} (mV)	Tafel slope (mV dec^{-1})	Ref
**FeCoNiMnRu/CNFs**	**145**	**61.3**	**This work**
FeCoNiPB/$(FeCoNi)_3O_{4-x}$	229	146	25
MnFeCoNi HEA	302	83.7	26
$Fe_{29}Co_{27}Ni_{23}Si_9B_{12}$ ribbon	230	85	27
CoCrFeNiMo-20 Mg	220	59.0	28
MCPS	288	27.7	29
$La(CrMnFeCo_2Ni)O_3$ HEPO	325	51.2	30
CoCuFeMoOOH@Cu	199	48.8	31
FeNiCoCrMn-G	229	40	5
HEF7	292	39	32
AlCrCuFeNi	270	77.5	33
$(Ru–Co)O_x$	171.2	60.8	7
Ru_1Ni_1-NCNFs	290	/	8
RuO_2/NiO/NF	250	50.5	10
Ru-NiFe-MOF/NF	205	50	34
$Ru_{SA}CoFe_2$/G	180	51	35
NiFe-MOF-5	168	42	14
Ru/NF-2	330	62	15
Ru-CoV-LDH/NF	230	81.2	24
Ru–NiFe LDH-F/NF	230.0	50.2	36
Co(S)-Fe(S)	355	62	37
Ni:Pi-Fe/NF	220	37	38
$Ni_2P/(NiFe)_2P(O)$ NAs	150	60	39
$Cu_{0.81}Co_{2.19}O_4$	290	65.6	40
NiFe/NF	191	44.1	41

Supplementary Table 8. Summarized overpotentials at 10 mA cm^{-2} (η_{10}) and Tafel slopes of electrocatalysts for OWS in 1.0 M KOH.

Electrocatalysts	cell voltage @ 10 mA cm^{-2}	Ref
**FeCoNiMnRu/CNFs**	**1.452**	**This work**
FeNiCoCrMn-G	1.63	5
$(Ru–Co)O_x$	1.488	7
Ru_1Ni_1-NCNFs	1.564	8
RuO_2/NiO/NF	1.5	10
Ru-NiFe-P	1.47	11
NiFe-MOF-5	1.57	14
Ru/NF-2	1.56	15
$Ru/Ni(OH)_2$/NF	1.50	20
Ru-CoV-LDH/NF	1.50	24

Supplementary Table 9. ΔG_{H*} of Fe-, Co-, Ni-, Ru-sites in FeCoNiMnRu with various atomic configurations.

Structure	Site	H Free Energy (eV)
Configuration 1	Fe	-0.22954
	Ni	-0.23571
	Co	-0.25067
	Ru	-0.21156
Configuration 2	Fe	-0.20215
	Ni	-0.30751
	Co	-0.20661
	Ru	-0.11792
Configuration 3	Fe	-0.25743
	Ni	-0.18365
	Co	-0.21365
	Ru	-0.21036
Configuration 4	Fe	-0.13054
	Ni	-0.27281
	Co	-0.43812
	Ru	-0.07115
Configuration 5	Fe	-0.32153
	Ni	-0.34089
	Co	-0.26261
	Ru	-0.25932
Configuration 6	Fe	-0.31425
	Ni	-0.21751
	Co	-0.20561
	Ru	-0.206913
Configuration 7	Fe	-0.305413
	Ni	-0.215841
	Co	-0.236172
	Ru	-0.213653
Configuration 8	Fe	-0.31423
	Ni	-0.25361
	Co	-0.21454
	Ru	-0.20361

Supplementary Table 10. ΔG_{H^*} of Fe-, Co-, Ni-, Ru-sites in FeCoNiCrRu with various atomic configurations.

Structure	Site	H Free Energy (eV)
Configuration 1	Fe	-0.29541
	Ni	-0.25816
	Co	-0.29615
	Ru	-0.26035
Configuration 2	Fe	-0.39654
	Ni	-0.33263
	Co	-0.31951
	Ru	-0.20592
Configuration 3	Fe	-0.32542
	Ni	-0.20654
	Co	-0.29234
	Ru	-0.26015
Configuration 4	Fe	-0.21879
	Ni	-0.34942
	Co	-0.60138
	Ru	-0.14727
Configuration 5	Fe	-0.30413
	Ni	-0.36581
	Co	-0.27156
	Ru	-0.26571
Configuration 6	Fe	-0.32474
	Ni	-0.25415
	Co	-0.26027
	Ru	-0.22941
Configuration 7	Fe	-0.341552
	Ni	-0.245913
	Co	-0.263512
	Ru	-0.289521
Configuration 8	Fe	-0.30355
	Ni	-0.26304
	Co	-0.20148
	Ru	-0.23651

Supplementary Table 11. ΔG_{H^*} of Fe-, Co-, Ni-, Ru-sites in FeCoNiCuRu with various atomic configurations.

Structure	Site	H Free Energy (eV)
Configuration 1	Fe	-0.3136
	Ni	-0.2715
	Co	-0.2815
	Ru	-0.2715
Configuration 2	Fe	-0.32695
	Ni	-0.37994
	Co	-0.38255
	Ru	-0.26513
Configuration 3	Fe	-0.31361
	Ni	-0.27156
	Co	-0.28154
	Ru	-0.26556
Configuration 4	Fe	-0.29566
	Ni	-0.40929
	Co	-0.52791
	Ru	-0.23708
Configuration 5	Fe	-0.32551
	Ni	-0.32692
	Co	-0.29647
	Ru	-0.28691
Configuration 6	Fe	-0.33691
	Ni	-0.27571
	Co	-0.29617
	Ru	-0.33654
Configuration 7	Fe	-0.35691
	Ni	-0.29657
	Co	-0.27891
	Ru	-0.28952
Configuration 8	Fe	-0.36694
	Ni	-0.22656
	Co	-0.29651
	Ru	-0.26015

Supplementary reference

1. Yao, R. et al. Nanoporous surface high-entropy alloys as highly efficient multisite electrocatalysts for nonacidic hydrogen evolution reaction. *Adv. Funct. Mater.* **31**, 2009613 (2021).
2. Jia, Z. et al. A novel multinary intermetallic as an active electrocatalyst for hydrogen evolution. *Adv. Mater.* **32**, 2000385 (2020).
3. Zhang, G. et al. High entropy alloy as a highly active and stable electrocatalyst for hydrogen evolution reaction. *Electrochim. Acta* **279**, 19-23 (2018).
4. Guo, J. et al. High electrocatalytic hydrogen evolution activity on a coupled Ru and CoO hybrid electrocatalyst. *J. Energy Chem.* **37**, 143-147 (2019).
5. Nguyen, T. X., Su, Y., Lin, C., Ruan, J. & Ting, J. A new high entropy glycerate for high performance oxygen evolution reaction. *Adv. Sci.* **8**, 2002446 (2021).
6. Jia, Z. et al. A self-supported high-entropy metallic glass with a nanosponge architecture for efficient hydrogen evolution under alkaline and acidic conditions. *Adv. Funct. Mater.* 2101586 (2021).
7. Wang, C. & Qi, L. Heterostructured inter-doped ruthenium–cobalt oxide hollow nanosheet arrays for highly efficient overall water splitting. *Angew. Chem. Int. Ed.* **59**, 1–7 (2020).
8. Li, M. et al. RuNi nanoparticles embedded in N-doped carbon nanofibers as a robust bifunctional catalyst for efficient overall water splitting. *Adv. Sci.* **7**, 1901833 (2020).
9. Xiao, X. et al. In situ growth of Ru nanoparticles on (Fe,Ni)$(OH)_2$ to boost hydrogen evolution activity at high current density in alkaline media. *Small Methods* **4**, 1900796 (2020).
10. Liu, J. et al. NiO as a bifunctional promoter for RuO_2 toward superior overall water splitting. *Small* **14**, 1704073 (2018).
11. Qu, M. et al. Regulating electron density of NiFe-P nanosheets electrocatalysts by a trifle of Ru for high-efficient overall water splitting. *Appl. Catal. B: Environ.* **263**, 118324 (2020).
12. Wang, D., Li, Q., Han, C., Xing, Z. & Yang, X. Single-atom ruthenium based catalyst for enhanced hydrogen evolution. *Appl. Catal. B: Environ.* **249**, 91-97 (2019).
13. Li, D. et al. Boosting hydrogen evolution activity in alkaline media with dispersed ruthenium clusters in NiCo-layered double hydroxide. *Electrochem. Commun.* **101**, 23-27 (2019).
14. Mou, Q. et al. A bimetal hierarchical layer structure MOF grown on Ni f foam as a bifunctional catalyst for the OER and HER. *Inorg. Chem. Front.* **8**, 2889 (2021).
15. Pei, Y. et al. Interface engineering with ultralow ruthenium loading for efficient water splitting. *ACS Appl. Mater. Interfaces* **12**, 36177−36185 (2020).
16. Zhang, H., Zhou, W., Lu, X. F., Chen, T. & Lou, X. W. Implanting isolated Ru atoms into edge-rich carbon matrix for efficient electrocatalytic hydrogen evolution. *Adv. Energy Mater.* **10**, 2000882 (2020).
17. Fan, L. et al. Ru nanoparticles encapsulated in ZIFs-derived porous N-doped hierarchical carbon nanofibers for enhanced hydrogen evolution reaction. *Catal. Sci. Technol.* **10**, 7302–7308 (2020).
18. Ding, R. et al. Salt template-assisted in situ construction of Ru nanoclusters and porous carbon: excellent catalysts toward hydrogen evolution, ammonia-borane hydrolysis, and 4-nitrophenol reduction. *Green Chem.* **22**, 835–842 (2020).
19. Fan, L. et al. Electrospun Ru–RuO_2/MoO_3 carbon nanorods with multi-active components: a Pt-like catalyst for the hydrogen evolution reaction. *Chem. Commun.* **56**, 739-742 (2020).
20. Chen, Q., Yang, X., Hou, C., Li, K. & Chen, Y. Inlay of ultrafine Ru nanoparticles into a self-supported $Ni(OH)_2$ nanoarray for hydrogen evolution with low overpotential and enhanced kinetics. *J. Mater. Chem. A* **7**, 11062–11068 (2019).
21. Xia, J. et al. Low-cost porous ruthenium layer deposited on Ni foam as a highly active universal-pH electrocatalyst for hydrogen evolution reaction. *ChemSusChem* **12**, 2780-2787 (2019).
22. Barman, B. K. et al. In situ decoration of ultrafine Ru nanocrystals on N-doped graphene tube and their applications as oxygen reduction and hydrogen evolution catalyst. *ACS Appl. Energy Mater.* **2**, 7330−7339 (2019).
23. Zhang, H. et al. Synergistic engineering of morphology and electronic structure in constructing metal-organic framework-derived Ru doped cobalt-nickel oxide heterostructure towards efficient alkaline hydrogen evolution reaction. *Chem. Eng. J.*

426, 131300 (2021).
24. Li, W., Feng, B., Yi, L., Li, J. & Hu, W. Highly efficient alkaline water splitting with Ru-doped Co-V layered double hydroxide nanosheets as bifunctional electrocatalyst. *ChemSusChem* 14, 730-737 (2021).
25. Wei, R. et al. Defect-rich FeCoNiPB/$(FeCoNi)_3O_{4-x}$ high-entropy composite nanoparticles for oxygen evolution reaction: impact of surface activation. *Appl. Surf. Sci.* **549**, 149327 (2021).
26. Dai, W., Lu, T. & Pan, Y. Novel and promising electrocatalyst for oxygen evolution reaction based on MnFeCoNi high entropy alloy. *J. Power Sources* **430**, 104-111 (2019).
27. Wang, H. et al. Nanostructured amorphous $Fe_{29}Co_{27}Ni_{23}Si_9B_{12}$ high-entropy-alloy: an efficient electrocatalyst for oxygen evolution reaction. *J. Mater. Sci. Technol.* **68**, 191-198 (2021).
28. Tang, J., Xu, J. L., Ye, Z. G., Li, X. B. & Luo, J. M. Microwave sintered porous CoCrFeNiMo high entropy alloy as an efficient electrocatalyst for alkaline oxygen evolution reaction. *J. Mater. Sci. Technol.* **79**, 171-177 (2021).
29. Ding, Z. et al. High entropy intermetallic–oxide core–shell nanostructure as superb oxygen evolution reaction catalyst. *Adv. Sustainable Syst.* **4**, 1900105 (2020).
30. Nguyen, T. X., Liao, Y., Lin, C., Su, Y. & Ting, J. Advanced high entropy perovskite oxide electrocatalyst for oxygen evolution reaction. *Adv. Funct. Mater.* **31**, 2101632 (2021).
31. Zhang, L., Cai, W. & Bao, N. Top-level design strategy to construct an advanced high-entropy Co–Cu–Fe–Mo (oxy)hydroxide electrocatalyst for the oxygen evolution reaction. *Adv. Mater.* **33**, 2100745 (2021).
32. Sukkurji, P. A. et al. Mechanochemical synthesis of novel rutile-type high entropy fluorides for electrocatalysis. *J. Mater. Chem. A* **9**, 8998–9009 (2021).
33. Liu, L., Li, N., Han, M., Han, J. & Liang, H. Scalable synthesis of nanoporous high entropy alloys for electrocatalytic oxygen evolution. Rare Met. (2021).
34. Lin, Y., Zhao, L., Wang, L. & Gong, Y. Ruthenium-doped NiFe-based metal–organic framework nanoparticles as highly efficient catalysts for the oxygen evolution reaction. *Dalton Trans.* **50**, 4280–4287 (2021).
35. Lee, J. et al. Stabilizing the OOH* intermediate via pre-adsorbed surface oxygen of a single Ru atom-bimetallic alloy for ultralow overpotential oxygen generation. *Energy Environ. Sci.* **13**, 5152-5164 (2020).
36. Wang, Y. et al. Interfacial synergy between dispersed Ru sub-nanoclusters and porous NiFe layered double hydroxide on accelerated overall water splitting by intermediate modulation. *Nanoscale* **12**, 9669–9679 (2020).
37. Maccato, C. et al. Plasma-assisted synthesis of Co_3O_4-based electrocatalysts on Ni foam substrates for the oxygen evolution reaction. *Adv. Mater. Interfaces* **8**, 2100763 (2021).
38. Li, Y. & Zhao, C. Iron-doped nickel phosphate as synergistic electrocatalyst for water oxidation. *Chem. Mater.* **28**, 5659−5666 (2016).
39. Xi, W. et al. Oxygen-doped nickel iron phosphide nanocube arrays grown on Ni foam for oxygen evolution electrocatalysis. *Small* **14**, 1802204 (2018).
40. Choi, W. et al. Three-dimensional honeycomb-like $Cu_{0.81}Co_{2.19}O_4$ nanosheet arrays supported by Ni foam and their high efficiency as oxygen evolution electrodes. *ACS Appl. Mater. Interfaces* **10**, 38663−38668 (2018).
41. Jin, J. et al. Exceptional electrocatalytic oxygen evolution efficiency and stability from electrodeposited NiFe alloy on Ni foam. *Electrochim. Acta* **299**, 567-574 (2019).
